# Systematically Revealing Quantitative Multi‐Target Integrative Effects of Plants With Artificial Intelligence Method

**DOI:** 10.1111/pbi.70321

**Published:** 2025-08-18

**Authors:** Jiang Qi‐yu, Ren Tian‐ai, Fan Xin‐yu, Zeng Hui‐yan

**Affiliations:** ^1^ Guangzhou University of Chinese Medicine Guangzhou Guangdong China; ^2^ Guangdong Provincial Hospital of Traditional Chinese Medicine Guangzhou Guangdong China; ^3^ State Key Laboratory of Dampness Syndrome of Chinese Medicine Guangzhou Guangdong China; ^4^ State Key Laboratory of Traditional Chinese Medicine Syndrome Guangzhou Guangdong China

**Keywords:** atherosclerosis, cell communication, immune infiltration, perivascular adipose tissue, Pseudotime difference analysis, single‐cell transcriptome artificial intelligence

## Abstract

Many plants have multiple chemical components and multiple targets, and their potential effects on diseases are the integrative effects of multiple targets. How to systematically reveal the integrated multi‐targets effect of plants on diseases is not only a challenge, but also an innovation. This study developed a novel research method based on artificial intelligence and took hawthorn as an example; a deep auto‐encoding neural network model was used to encode the expression levels of multiple common targets between hawthorn and atherosclerosis in each cell of the single‐cell transcriptome of atherosclerotic perivascular adipose tissue (PVAT) as an integrated value (MTIS). The landscape and quantitative mapping of multi‐targets potential integrated effect of plants on disease at the single‐cell level would be achieved based on this innovative approach, and in‐depth analysis such as MTIS comparisons, MTIS‐pseudotime difference analysis, cell communication analysis, and immune infiltration analysis, was performed to reveal the potential mechanism and landscapes of hawthorn on the PVAT microenvironment of atherosclerotic. Due to many plants for disease having multiple chemical compositions and multiple targets, the novel method proposed in this study may have a wide range of applications.

## 1 Introduction

Many plants have multiple chemical components and targets, and their potential effects on diseases are the result of the integrative effects of multiple targets. Systematically revealing the integrated multitarget effects of plants on diseases is not only a challenge but also an innovation. Currently, research on the effects of plants on diseases is mostly focused on experimental research. With the development of artificial intelligence technology, its application in biomedical analysis is becoming increasingly widespread (Warraich et al. 2024; Farnoosh and Abnoosian 2024; Abnoosian et al. 2023a, 2023b). Recently, deep autoencoding neural network models have been applied to the processing of cell and gene data (Tang et al. 2024; Avchaciov et al. 2022). In recent years, the application of single‐cell transcriptome technologies has enabled the elucidation of biological mechanisms at the single‐cell level (Wen et al. 2022). This study used hawthorn as an example and proposed a novel research method to reveal the potential mechanisms of action of hawthorn in the PVAT microenvironment of atherosclerosis based on artificial intelligence.

Hawthorn is a delicious food that is beloved by many people. It is not only a fruit but also a potential drug that can improve atherosclerosis. Some studies have shown that hawthorn can reduce blood lipid levels, soften blood vessels, and slow the progression of atherosclerosis (Lu et al. 2023). Atherosclerosis is a progressive disorder that can lead to serious cardiovascular and cerebrovascular diseases such as myocardial infarction and stroke. The increase in low‐density lipoprotein levels in the blood vessels leads to an increase in oxidised LDL (OX‐LDL), which has an inflammatory immune reaction with the vascular endothelium, causing damage to the vascular endothelium. Foam cells that swallow OX‐LDL continuously deposit under the vascular intimae, which then produces fibrous plaques and forms atherosclerosis (Patterson et al. 2024). In recent years, some studies have found that the perivascular adipose tissue (PVAT) of atherosclerosis has a key impact on the process of atherosclerosis, and that there are interactions between PVAT and the vascular environment of atherosclerosis (Balakumar et al. 2024; Qi et al. 2018). Although there have been many studies on the mechanisms of hawthorn in atherosclerosis (Lu et al. 2023), the mechanism by which it affects PVAT in atherosclerosis remains unclear. Because the essence of atherosclerosis is the immune inflammatory reaction between lipid molecules and the vascular endothelium, exploring the potential mechanism of hawthorn on the PVAT immune microenvironment of atherosclerosis is a new research topic. More importantly, the effects of plants are often attributed to the combined effects of multiple targets, revealing that the landscape and quantitative mapping of potential multitarget effects of plants on diseases at the single‐cell level is a challenge and innovation.

**FIGURE 1:**
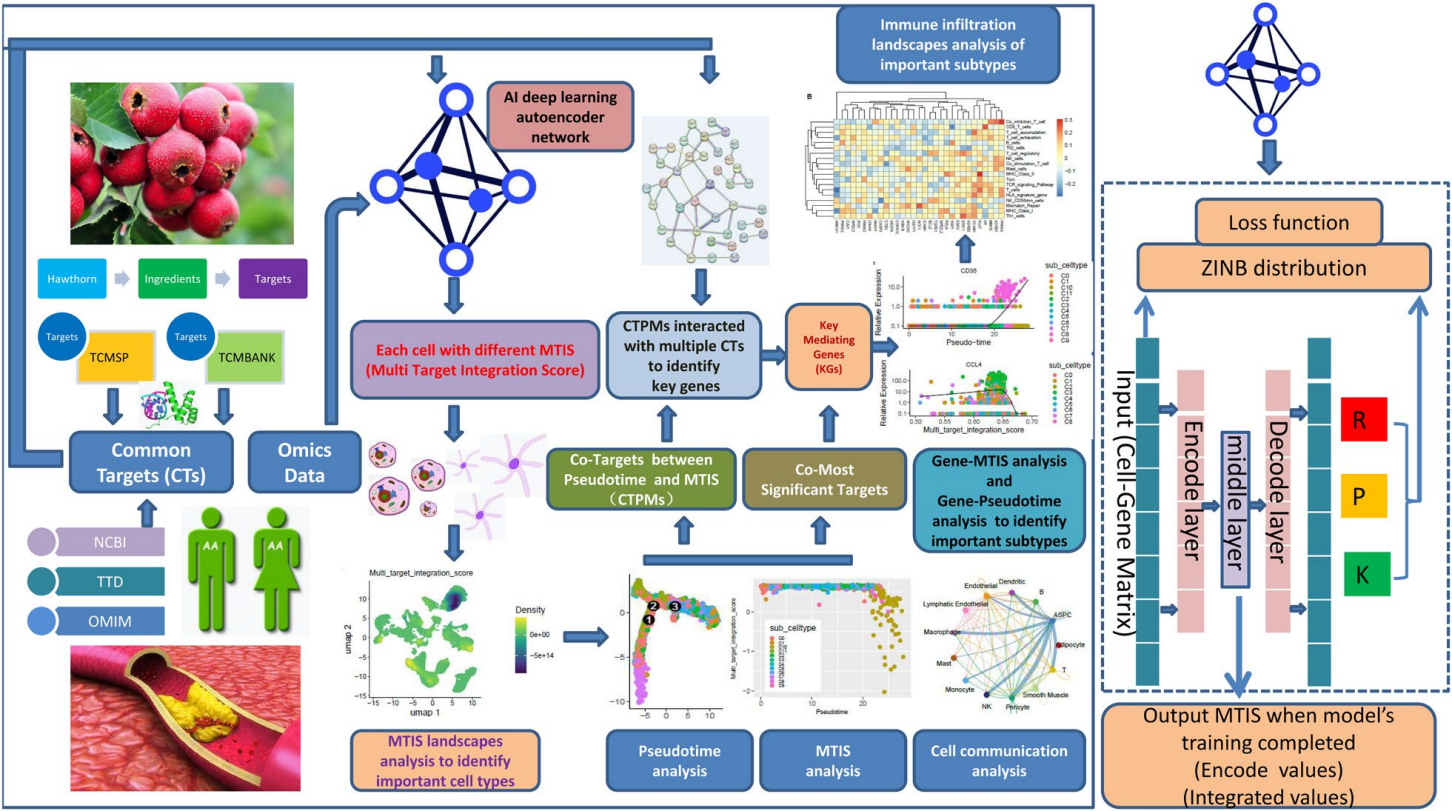
The framework of this study.

Intelligent data‐encoding and denoising technologies have been developed in recent years (Norozpour and Darbandi 2020; Darbandi 2017a, 2017b) In this study, a deep auto‐encoding neural network model (Eraslan et al. 2019) was used to encode the expression levels of multiple common targets between hawthorn and atherosclerosis in each cell of the single cell transcriptome of atherosclerotic PVAT as an integrated value. The landscape and quantitative mapping of multi‐targets potential integrated effect of plants on disease at the single cell level would be achieved based on this innovative approach, and in‐depth analysis such as MTIS comparisons, MTIS‐pseudo‐time difference analysis, cell communication analysis, and immune infiltration analysis were performed to reveal the potential mechanism and landscapes of hawthorn on the PVAT microenvironment of atherosclerotic. The detailed research framework is shown in Figure 1.

## 2 Method

### 2.1 Data Collection

As shown in Figure 1, we first collected the data on the active ingredients of hawthorn fruits and leaves from the TCMSP (Traditional Chinese Medicine Systems Pharmacology) (Ru et al. 2014) and TCMBANK (Lv et al. 2023) databases, as well as the targets of the active ingredients. The targets of atherosclerosis were collected from (National Center for Biotechnology Information) database (Schoch et al. 2020), TTD (Therapeutic Target Database) database (Zhou et al. 2024), and OMIM (Online Mendelian Inheritance in Man) database (Hamosh et al. 2021). Common targets (CTs) between hawthorn and atherosclerosis were used for further analysis. Three groups of atherosclerotic PVATs were collected from GSE233870 (Fu et al. 2023) in the GEO database (https://www.ncbi.nlm.nih.gov/geo/query/acc.cgi?acc=GSE233870). The three groups of samples were subjected to single‐cell RNA sequencing of the coronary artery PVATs in the early and advanced stages of human atherosclerosis, as well as in the control group. The control group underwent single‐cell RNA sequencing of coronary artery PVAT without atherosclerosis. Raw sequence data was provided at China Genomic Sequence Archive (GSA) (Fu et al. 2023; Wang et al. 2017). The expression data of CTs in all the cells of the three single‐cell transcriptomes were extracted and used as inputs for the deep auto‐encoding artificial intelligence model.

### 2.2 Model Training and Encoding

There are many different machine learning models in the field of artificial intelligence, such as graph neural networks and Bayesian methods. Graph neural networks can predict the classification of nodes or graphs based on the feature vectors of nodes and the topological relationships between nodes, whereas Bayesian methods can predict the classification of outcome variables based on data from multiple input variables. The commonality of these machine learning models is that they predict the classification of outcome variables based on multidimensional input variables; this study required a machine learning method that could encode (dimension‐reduction) multidimensional variables into one‐dimensional variables. Therefore, a deep autoencoding neural network was a suitable model for this study. The framework of the deep learning autoencoding neural network (Eraslan et al. 2019) used in this study, which consists of five layers, is shown on the right panel of Figure 1. The first layer was the input layer, the cell‐gene matrix was input into the layer, and the cell‐gene matrix was a subset of single‐cell transcriptome data that included only the counts (expression levels) of genes in CTs. If there were N cells in the single‐cell transcriptome data and Q genes in the CTs, the size of the input cell‐gene matrix was N multiplied by Q. The cell‐gene matrix was processed by neurons and transmitted to the second layer. The second layer was the encoding layer with 64 neurons, which was responsible for reducing (encoding) high‐dimensional CT data into low‐dimensional representations. The third layer was the middle layer with one neuron. The one‐dimensional encoded value (integrated value) generated by this layer was called the Multi‐Target Integration Score (MTIS) and was used for further analysis when the model training was completed. The fourth layer was the decoding layer, which would decode in reverse based on the encoded values of the third layer in an attempt to recover high‐dimensional CT data. The number of neurons in each layer was 64. The fifth layer was the output layer, which restored the high‐dimensional data of CTs. Usually, this layer output denoising high‐dimensional data of CTs, rather than the original input data, and it also output the three parameter matrices R, K, and P of the zero‐inflation negative binomial (ZINB) distribution (Eraslan et al. 2019).

The ZINB distribution is a statistical distribution model developed based on a negative binomial distribution that is suitable for data with many zero values. Single‐cell sequencing data often contain a large number of gene counts with zero values; therefore, compared with other statistical distribution models, the ZINB distribution is suitable for processing single‐cell sequencing data (Eraslan et al. 2019).

The training process of the model was as follows: First, the original cell‐gene matrix was input to the first layer and encoded by the encoding layer for each cell, where a 64‐dimensional variable represented the encoded value of a cell; that is, the expression levels of multiple genes in a single cell in the input cell‐gene matrix were represented as 64‐dimensional variables. In the middle layer, lower‐dimensional encoded values (integrated values) were generated for each cell, and a one‐dimensional variable represented the encoded value of a cell. Second, the encoded values (integrated values) were decoded by the decoding layer to generate the decoded values for each cell. At this moment, 64‐dimensional variables were used again to represent the encoded value of a cell. Starting from here, it would attempt to recover the original cell‐gene matrix from low‐ to high‐dimensional representations. The calculation formula for this process is shown in Equation (1), where CG represents the input cell‐gene matrix, Dc represents the decoded values, ReLU is the activation function in the neural network, *f*_norm_ is a normalisation function, and *W*_Ec_, *W*_M_, and *W*_Dc_ are the weight matrices of the neurons in the encoding, intermediate, and decoding layers, respectively. Third, the decoded value Dc was used to calculate the three parameter matrices R, P, and K of the ZINB distribution with Formula (2), where *W*_r_, *W*_p_, and *W*_k_ are the weight matrices of these three parameters in the neurons, respectively. The sigmoid is an activation function in neural networks, Tc is the total number of genes in each cell, and Mc is the median gene count for each cell. Fourth, according to Formula (3), R, P, and K were the parameters of the ZINB distribution, and the original input cell‐gene matrix CG was used as the observation data to calculate the likelihood of the ZINB distribution. The likelihood of the ZINB distribution formed the basis for the loss function.

During the training process, based on the ZINB distribution, the model compares the output data of the fifth layer with the original input data to calculate the loss of the model and adjusts the connection weights of all neurons at a certain learning rate. With the weights of the neurons continuously adjusted, the difference (loss value) between the output data of the model and original input data would converge according to a certain gradient. In other words, when the output data of the model approaches the input data, the encoded values of the third layer could more accurately represent the original expression data of the CTs. When the loss value was less than the set threshold, the training of the model ended, indicating that the encoding values of the third layer were able to accurately represent the gene expression data of the CTs at this time; thus, the encoding values of the third layer could be output as MTIS for further analysis in this study when the model's training was completed. The training parameters for the model were set as follows: the training epochs for this model were set to 300; the learning rate was set to 0.001; and the numbers of neurons in the encoding, intermediate, and decoding layers were set to 64, 1, and 64, respectively. The number of neurons in the input layer was the same as the number of targets in CTs.

The abovementioned deep learning autoencoder model has been proposed by scholars, used for denoising single‐cell transcriptome data (Eraslan et al. 2019), and further used for predicting neuronal activity. It encodes the expression data of multiple genes related to neuronal activity by using the model to generate integrated score values to characterise the neuronal activity of cells (Bahl et al. 2024). Therefore, based on the principle of similarity, this study encoded the expression data of multiple CTs using this model to generate integrated score values that were used to characterise the potential effects and analyse the potential mechanisms of action of hawthorn on the PVAT microenvironment of atherosclerosis. This analysis was conducted using Python.

(1) $$\mathrm{Dc}=\text{ReLU}\left(\text{ReLU}\left(\text{ReLU}\left(f_{\mathrm{norm}}\left(\mathrm{CG}\right)W_{\mathrm{Ec}}\right)W_{M}\right)W_{\mathrm{Dc}}\right)$$

(2) $$R=e^{{\mathrm{DcW}}_{r}},P=e^{{\mathrm{DcW}}_{p}},K=\frac{\mathrm{Tc}}{\mathrm{Mc}}\text{sigmoid}\left({\mathrm{DcW}}_{k}\right)$$

(3) $$\text{Loss}=f\left(\text{ZINB}\left(\mathrm{CG};K,R,P\right)\right)$$

### 2.3 Data Analysis

Three groups of single‐cell transcriptome data were processed using the Seurat pipeline (Mangiola et al. 2021). First, during the process of creating the Seurat object, the min.cells parameter and min.features parameter were set to 3 and 200, respectively, with the CreateSeuratObject function used to preliminarily screen qualified cells, and the remaining parameters were set to default. This ensured that each cell included in the study had at least 200 genes and that each gene existed in at least three cells. The PercentageEigenSet function was used to remove cells that might have died, with parameters similar to those used in traditional research. After completing the cell screening steps, all samples were merged into the same group. Then, the SCTransform function was employed for the normalisation of gene expression data to reach a unified metric for all data and to reduce batch effects; all parameters were default. The SCTransform function integrates NormalizeData, FindVariableFeatures, and ScaleData. In the traditional analysis steps, NormalizeData is used to correct for batch effects, FindVariableFeatures is used for variable feature selection, and ScaleData is used for data standardisation and scaling. The SCTransform function integrates the above three functions and can achieve the standardised preprocessing of single‐cell data in one step. To perform cell clustering, the PCA algorithm was used to calculate the principal components of single‐cell data, and the FindNeighbors algorithm was used to find neighbours between similar cells based on the top ten principal components. The FindClusters algorithm was used to cluster cells with a resolution parameter of 0.5. Finally, the RunUMAP function was used to reduce the dimensionality for two‐dimensional visualisation. The major cell types and subtypes were annotated with the Azimuth package. The analysis was conducted using the R language.

After inputting the RNA transcription data of each cell into a deep learning auto‐encoding neural network, the MTIS of each cell was obtained. To test whether the MTIS could reflect the potential mechanism of atherosclerosis, the mean MTIS of the above three groups of single‐cell transcriptome data was compared using the Wilcoxon test, and comparisons with *p*‐values less than 0.05 were considered statistically significant. Due to the uncertainty of whether the data morphology followed a normal distribution, the Wilcoxon test was used. Before comparison, all data were normalised using a scale function to align and control for potential confounding factors. MTIS included integrated scores of the expression levels of common targets between hawthorn and atherosclerosis. A significant difference between the mean MTIS of the non‐atherosclerotic group and the mean MTIS of the atherosclerotic group indicated that the MTIS was an indicator of the difference in hawthorn's effect on atherosclerotic vs. non‐atherosclerotic cells, which could reflect the potential mechanism of atherosclerosis to a certain extent. In addition, to determine which cells had differences in MTIS among the three groups, the mean MTIS between different cell types in the three groups was compared using the Wilcoxon test, and the cumulative sum of MTIS was compared intuitively. To further determine which cell types had significant differences in the distribution of MTIS between the non‐atherosclerotic and atherosclerotic groups, distribution maps of different cell types on cell clusters were plotted for the three groups of single‐cell transcriptome data. Further, the landscapes of density distributions of MTIS on cell clusters were also plotted and compared with the distribution maps of different cell types on cell clusters. The MTIS‐differential cell types (MTISDCT) between the atherosclerotic group and non‐atherosclerotic group, as determined by the above two analyses, were further used for the following analysis.

If a gene is important in atherosclerosis, it may exhibit different expression levels at different stages. To explore the potential mechanism of hawthorn in the PVAT microenvironment of atherosclerosis, pseudo‐time analysis of immune cells in MTISDCT (ICIM) was performed using the Monocle 2 package (Qiu et al. 2017). Pseudo‐time analysis could cluster cells based on the reverse graph embedding algorithm (Qiu et al. 2017) and generate pseudo‐time trajectories of cells. Pseudo‐time trajectory maps of the cell types, pseudo‐time values, and MTIS were plotted. The two‐dimensional variation relationships between the MTIS and pseudo‐time of each cell in the different cell types were plotted. To understand which gene expression levels varied with changes in pseudo‐time and MTIS values, heat maps of the top 50 differentially expressed genes that varied with pseudo‐time values, as well as the heat maps of the top 50 differentially expressed genes that varied with MTIS, were plotted. Common targets between the pseudo‐time‐ and MTIS‐differential gene analyses (CTPMs) were used for further analysis. To obtain the most significant common targets between pseudo‐time and MTIS variation analyses (co‐most significant targets), the two‐dimensional variation relationships between the *q* values of the top 50 differentially expressed genes that varied with MTIS and those that varied with pseudo‐time were also plotted. The interaction networks between CTPMs and CTs were generated with a parameter of high confidence score in the STRING database, and the interaction scores between targets greater than or equal to 0.7 were considered as high confidence scores in the STRING database (Szklarczyk et al. 2023). CTPMs interacting with at least two CTs, as well as co‐most significant targets, were considered key mediating genes (KGs) for the effects of hawthorn on the PVAT microenvironment of atherosclerosis. Furthermore, graphs of the expression levels of KGs in different cell types that varied with pseudo‐time and MTIS were generated to confirm the key cell subtypes. To identify key targets related to immunity (KTRI), immune infiltration landscape analysis of key subtypes was performed using the ssGSEA algorithm (Zhou et al. 2023). The correlation between the expression level of the target and millimetre microenvironment signals was analysed using Pearson's chi‐squared test. Targets with coefficients most correlated with immune microenvironment signatures and *p*‐values less than 0.05 in multiple immune infiltration landscape maps simultaneously were considered as KTRI. The density distribution of KTRI in various cell types was compared among the three groups using single‐cell transcriptome data.

In addition, non‐immune cells in MTISDCT (NICIM) may play a role in the microenvironment. To analyse the immune effects of NICIM in the potential mechanism of hawthorn on atherosclerotic PVAT, cellular communication between NICIM and other immune cells was analysed using the CellChat package (Jin et al. 2024). Firstly, the computeCommunProbPathway function was used to calculate the communication probabilities of various pathway levels between cell communications, and the netVisualbubble function was used to obtain pathways with a *p*‐value of less than or equal to 0.05 for communications between NICIM and other cells, and the gene names of receptors and ligands (LRNs) were obtained in these pathways. Second, the interaction network between CTs and LRNs was generated in the STRING database as a parameter with a high confidence level for interaction scores, and LRNs that interacted with at least two CTs were considered key LRNs. Third, the ligand‐receptor pairs of pathways containing key LRNs (key LRN pairs) were extracted using the extractEnrichdL function. The communication probabilities and *p*‐values of pathways enriched in key LRN pairs for communications of various cell types towards NICIM were calculated, and pathways with *p*‐values less than or equal to 0.05 are displayed in the graphs. Three groups of single‐cell transcriptome data were analysed using the above three steps. Compared with the non‐atherosclerotic group, the increased communication pathways in the other two atherosclerotic groups were key communication pathways (KCP). The strengths of communication between NICIM and other cells in the KCP in the early and advanced atherosclerosis groups are plotted. The expression levels of the genes involved in KCP in various cell types were also compared.

### 2.4 Model Evaluation

To further validate the reliability of the deep learning autoencoding neural network and demonstrate whether the integrated MTIS values of this model could accurately represent the expression levels of multiple targets in single cells, we compared three benchmark models with the deep learning autoencoding neural network. We used three single‐cell transcriptome datasets of PVAT in this study, specifically three subsets that only contained the expression levels of genes in CTs, as the experimental dataset, and normalised them with a scale function. The first group had advanced atherosclerosis (Advanced Group), the second group had early‐stage atherosclerosis (Early Group), and the third group did not have atherosclerosis (Non‐atherosclerosis group). In the first experiment, a deep learning auto‐encoding neural network was used to generate an MTIS value for each cell in the three groups mentioned above. The MTIS was used as a single‐dimensional input variable, whereas the group in which the cell was located was used as the label variable (outcome variable). Two well‐known algorithms in the field of machine learning, Random Forest (RF) and Extreme Radiant Boosting (XGBOOST), were used for training and prediction. The ROC curves were plotted to calculate the area under the ROC curve (AUC values). Fifty percent of the data in the three groups were randomly selected as training data, and the remaining data were used as testing data. The ‘ntree’ parameter of RF was set to 10, and the ‘max_depth’ parameter of XGBOOST was set to 3, and all other parameters were set to default values. In the second experiment, the expression levels of multiple genes in each cell were used as multidimensional input variables. The testing method and parameter settings were identical to those used in the first experiment. In the third experiment, the sum of the expression levels of multiple genes in each cell was used as a single‐dimensional input variable, and the testing method and parameter settings were the same as those in the first experiment. In the fourth experiment, the expression levels of multiple genes in each cell were processed using the UMAP algorithm to generate two‐dimensional variables for each cell representing the expression levels of multiple genes. The two‐dimensional variable of each cell was used as a two‐dimensional input variable, and the testing method and parameter settings were identical to those used in the first experiment. The ROC curves and AUC values of these four experiments were compared to evaluate whether the deep learning auto‐encoding neural network had sufficient advantages compared to the baseline models and whether the integrated MTIS values generated by the deep learning auto‐encoding neural network could accurately represent the expression levels of multiple targets in single cells.

### 2.5 Experiment Validation

Because of the involvement of multiple targets, based on the above findings, some key representative targets were selected for validation in this study, including CD36, CTSD, CCL3, IL1B, PPARG, and MT1X. These targets are involved in the formation of foam cells, vascular inflammation, fat regulation, and oxidative stress during atherosclerosis. This study was approved by the Ethics Committee of the Guangdong Provincial Hospital of Traditional Chinese Medicine.

#### 2.5.1 Cell Culture

##### 2.5.1.1 Murine Aortic Smooth Muscle Cells (MOVAS Cells)

MOVAS cells (EallBio Biomedical Technology Co. Ltd., Beijing, China) were maintained in T25 culture flasks with DMEM/H supplemented with 10% FBS, 1% antibiotics, and 0.2 mg/mL G418. They were maintained at 37°C in a 95% O_2_ and 5% CO_2_ atmosphere until reaching 90% confluence.

##### 2.5.1.2 Murine Embryo Fibroblasts (3T3‐L1 Cells)

3T3‐L1 cells are a mouse preadipocyte line widely used in the study of adipobiology, metabolic diseases, and molecular mechanisms because of their ability to differentiate into mature adipocytes. 3T3‐L1 cells, derived from 3T3 cells (Swissalbino), were cultured in T25 culture flasks in DMEM/H, 10% FBS, and 1% penicillin–streptomycin solution (P/S) at 37°C under a humidified atmosphere of 95% O_2_ and 5% CO_2_ to 90% confluence.

##### 2.5.1.3 Co‐Culture of MOVAS and 3T3‐L1 Cells

Co‐culture of MOVAS and 3T3‐L1 cells was used to simulate perivascular adipose tissue, and the supernatant collected from MOVAS cells after modelling was co‐cultured with 3T3 cells for 24 h.

##### 2.5.1.4 Experimental Groups

To verify the effect of hawthorn on atherosclerosis in the perivascular adipose tissue microenvironment, the experimental groups were established as follows: ① control group: MOVAS cells received medium without ox‐LDL, co‐cultured with 3T3 cells; ② model group: MOVAS cells were treated with ox‐LDL (2.0 mg/mL), co‐cultured with 3T3 cells; and ③ experimental groups: the remaining three experimental groups were exposed to conditioned medium containing varying concentrations of hawthorn extract (0.2 g·L^−1^, 0.4 g·L^−1^, 0.8 g·L^−1^) alongside ox‐LDL (2.0 mg/mL), co‐cultured with 3T3 and MOVAS cells. The medium was refreshed every 2 days throughout the experiment.

#### 2.5.2 Triglyceride (TG) Assay

The prepared MOVAS cell suspension was centrifuged at 1000 rpm for 10 min; the supernatant was discarded, and the cell pellet was retained. The cells were then washed once with 0.1 mol/L phosphate buffer (pH 7–7.4) and centrifuged again at 1000 rpm for 10 min. Afterward, 0.2 mL of normal saline was added to the cell pellet for homogenization, followed by sonication in an ice water bath at 300 W for 3–5 s, with 30‐s intervals, repeated twice. The absorbance of the homogenate was measured, and triglyceride (TG) concentrations were calculated.

#### 2.5.3 Quantitative Real‐Time PCR (qRT‐PCR)

Total mRNA was extracted from cells in each group using ABclonal 2X Universal SYBR Green Fast qPCR Mix reagent, followed by reverse transcription to cDNA, according to the manufacturer's protocol. Primers for the target genes were synthesised by Tsingke Biotechnology Co. Ltd. The primer sequences are listed in Table 1. The selected targets included: ① CD36, ② CTSD, ③ CCL3, ④ IL1B, ⑤ PPARG, ⑥ MT1X. mRNA expression levels of these genes were assessed using RT‐qPCR, with amplification cycles set at 95°C for 5 s and 60°C for 30 s over 40 cycles. Data analysis was conducted using the 2^−△△CT^ method. RT‐qPCR was performed in duplicate using cDNA from five separate cell cultures.

**TABLE 1:** Primer sequences of PCR.

Gene	Forward (5′ → 3′)	Reverse (5′ → 3′)
CD36	ATGGGCTGTGATCGGAACTG	TTTGCCACGTCATCTGGGTTT
CTSD	GCTTCCGGTCTTTGACAACCT	CACCAAGCATTAGTTCTCCTCC
CCL3	TGTACCATGACACTCTGCAAC	CAACGATGAATTGGCGTGGAA
IL1B	TTCAGGCAGGCAGTATCACTC	GAAGGTCCACGGGAAAGACAC
PPARG	GGAAGACCACTCGCATTCCTT	GTAATCAGCAACCATTGGGTCA
MT1X	GTGAGGTGTGTATAGATGTGGGG	ACGTCTTGCTGAGGTAACCTG
Actin	CCCAAAGCTAACCGGGAGAAG	GACAGCACCGCCTGGATAG

#### 2.5.4 Statistical Analysis

Statistical analysis was performed using the SPSS 25.0 software. ANOVA was used to assess statistical significance, with *p* < 0.05 considered indicative of a significant difference. First, the Shapiro Wilk's test was used to determine whether the data followed normal distributions; then, Levene's test was used to determine homogeneity of variance, and finally, the ANOVA analysis was used for statistical difference testing.

## 3 Result

### 3.1 Data Collection

A total of 139 target data of hawthorn were collected from the TCMSP database (detailed in File S1), 13 hawthorn targets were collected from the TCMBANK database (detailed in File S2), and 1295 targets related to atherosclerosis were collected from the NCBI, TTD, and OMIM databases (detailed in File S3). In the above collection, we focused only on targets of the human species. A total of 48 common targets (CTs) between hawthorn and atherosclerosis were identified (File S4). The CTs included GSTM1, IL1A, CAV1, HMOX1, GSTP1, XDH, CDKN1C, IGF2, IL1B, and MPO. In the GSE233870 dataset, the numbers of samples in the non‐atherosclerosis, early atherosclerosis, and advanced atherosclerosis groups were four, three, and three, respectively. The expression of CTs in the non‐atherosclerosis, early stage atherosclerosis, and advanced atherosclerosis groups was processed using a deep auto‐encoding neural network model and was generated (encoded) as MTIS for each cell (detailed in Files S5–S7). The gene expression data of 48 CTs from the three single‐cell transcriptomes mentioned above were input into a deep learning auto‐encoding neural network to generate the MTIS for each cell.

### 3.2 Comparisons of MTIS of Single Cell Transcriptome Data Between Non‐Atherosclerosis and Atherosclerosis Groups

**FIGURE 2:**
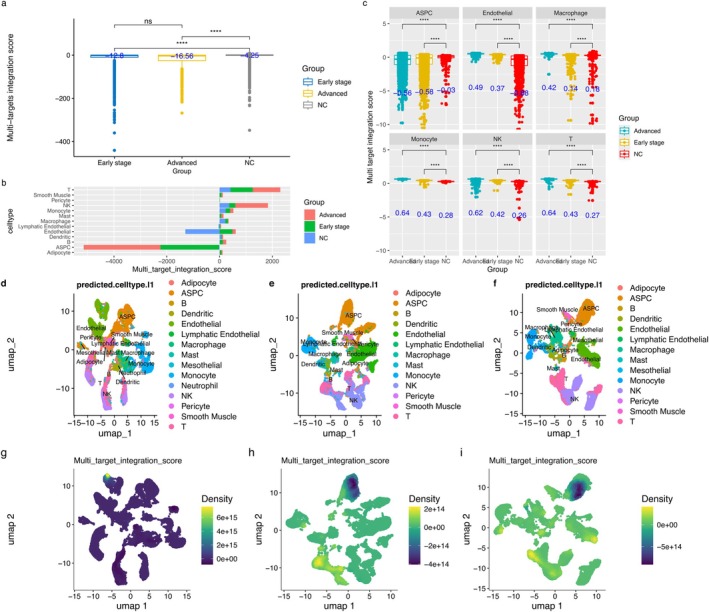
(a) Mean comparisons of MTIS between non‐atherosclerosis group, early atherosclerosis group and advanced atherosclerosis group. (b) Accumulated values of MTIS in different cells between non‐atherosclerosis group, early atherosclerosis group and advanced atherosclerosis group. (c) Mean comparisons of MTIS in different cells between non‐atherosclerosis group, early atherosclerosis group and advanced atherosclerosis group. (d) Landscapes of different cell distributions in non‐atherosclerosis group. (e) Landscapes of different cell distributions in advanced atherosclerosis group. (f) Landscapes of different cell distributions in early atherosclerosis group. (g) Landscapes of MTIS distributions in non‐atherosclerosis group. (h) Landscapes of MTIS distributions in advanced atherosclerosis group. (i) Landscapes of MTIS distributions in early atherosclerosis group.

The mean comparisons of MTIS between the non‐atherosclerosis, early atherosclerosis, and advanced atherosclerosis groups are shown in Figure 2a. There were significant differences between the non‐atherosclerosis and early atherosclerosis groups, and between the non‐atherosclerosis and advanced atherosclerosis groups (*p* < 0.001). As MTIS could reflect the differences in the effects of hawthorn on the non‐atherosclerosis group and atherosclerosis group, the following analysis based on MTIS could reveal the potential mechanisms of hawthorn on the atherosclerosis group. The non‐atherosclerotic group (NC group) had the maximum mean value of −4.25, while the latter group had a minimum mean value of −16.56. The MTIS values decreased as the disease progressed. In Figure 2, the differences in cumulative MTIS values for various cell types are shown in Figure 2b. Compared with the NC group, ASPC were clearly different in the early‐ and advanced‐stage groups, indicating that CTs might mainly infiltrate ASPC cells. The endothelial cells in the NC group were negative, whereas those in the early‐ and advanced‐stage groups were positive, indicating that the difference in MTIS in endothelial cells could distinguish the NC group from the atherosclerosis group. In addition, T cells in the NC group were significantly different from those in the early‐ and advanced‐stage groups. This indicates that understanding the potential mechanisms of hawthorn in some cell types is worthy of further exploration. In Figure 2c, further comparisons of the mean of MTIS among various major cell types showed that there were differences between the NC group and the early stage and advanced groups across all major cell types (*p* < 0.001). Among adipose stem cells and progenitor cells (ASPCs), the NC group had the maximum value, whereas in most other cell types, the NC group had the minimum value. Figure 2d–i show the landscapes of different cell distributions and landscapes of different MTIS distributions in the three groups. According to Figure 2d,g, the bright areas in Figure 2g (NC group) represent endothelial cells. Similarly, according to Figure 2e,h, the bright areas in Figure 2h (advanced group) represent T and NK cells, while the dark areas represent ASPC. According to Figure 2f,i, it could be seen that the bright areas in Figure 2i (early‐stage group) were T cells, endothelial cells, and monocytes, and the dark areas were ASPC cells. Furthermore, compared to Figure 2g (NC group), the most significant differences in Figure 2h (advanced group) and Figure 2i (early‐stage group) were observed in T cells, NK cells, ASPC cells, and monocytes. Considering the analysis results of Figure 2b,c,g,h,i, T cells in the advanced group, NK cells in the advanced group, monocytes in the early stage group, and ASPC cells were used for further analysis as MTIS‐differential cell types (MTISDCT). T cells, NK cells, and monocytes are immune cells of the MTIS‐differential cell type (ICIM), and ASPC are non‐immune cells of the MTIS‐differential cell type (NICIM).

### 3.3 Pseudo‐Time‐MTIS Analysis of Immune Cells in the MTIS‐Differential Cell Types

#### 3.3.1 Analysis of NK Cells in Advanced Atherosclerosis

**FIGURE 3:**
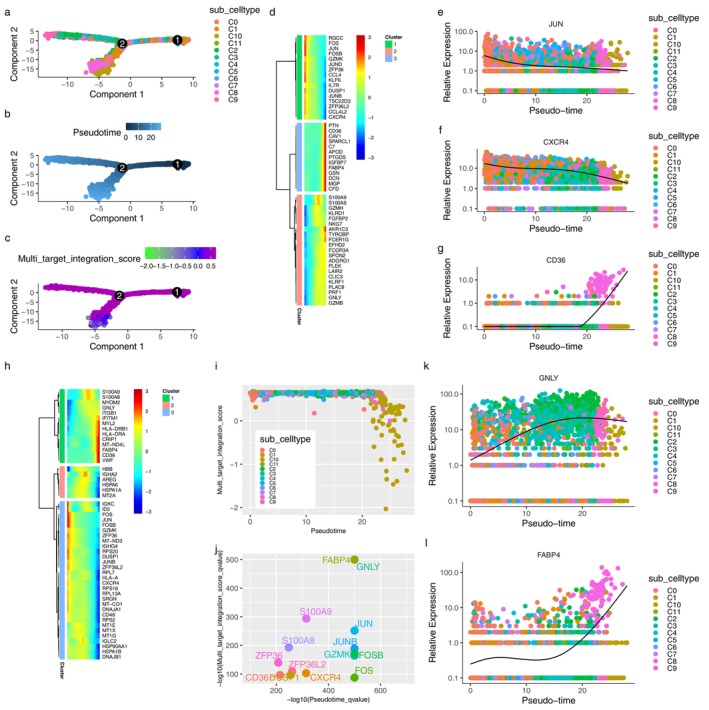
(a) Distributions of NK cell subtypes in trajectory analysis. (b) Distributions of pseudotime in trajectory analysis. (c) Distributions of MTIS in trajectory analysis. (d) Heatmap of the top 50 differentially expressed genes with pseudotime changes. (h) Heatmap of the top 50 differentially expressed genes with MTIS changes. (e) Expressions of JUN in various cell subtypes with pseudotime changes. (f) Expressions of CXCR4 in various cell subtypes with pseudotime changes. (g) Expressions of CD36 in various cell subtypes with pseudotime changes. (k) Expressions of GNLY in various cell subtypes with pseudotime changes. (l) Expressions of FABP4 in various cell subtypes with pseudotime changes. (i) Two dimensional relationships of cells between pseudotime and MTIS. (j) Two dimensional relationships of genes between significant *q*‐values of pseudotime and MTIS.

**FIGURE 4:**
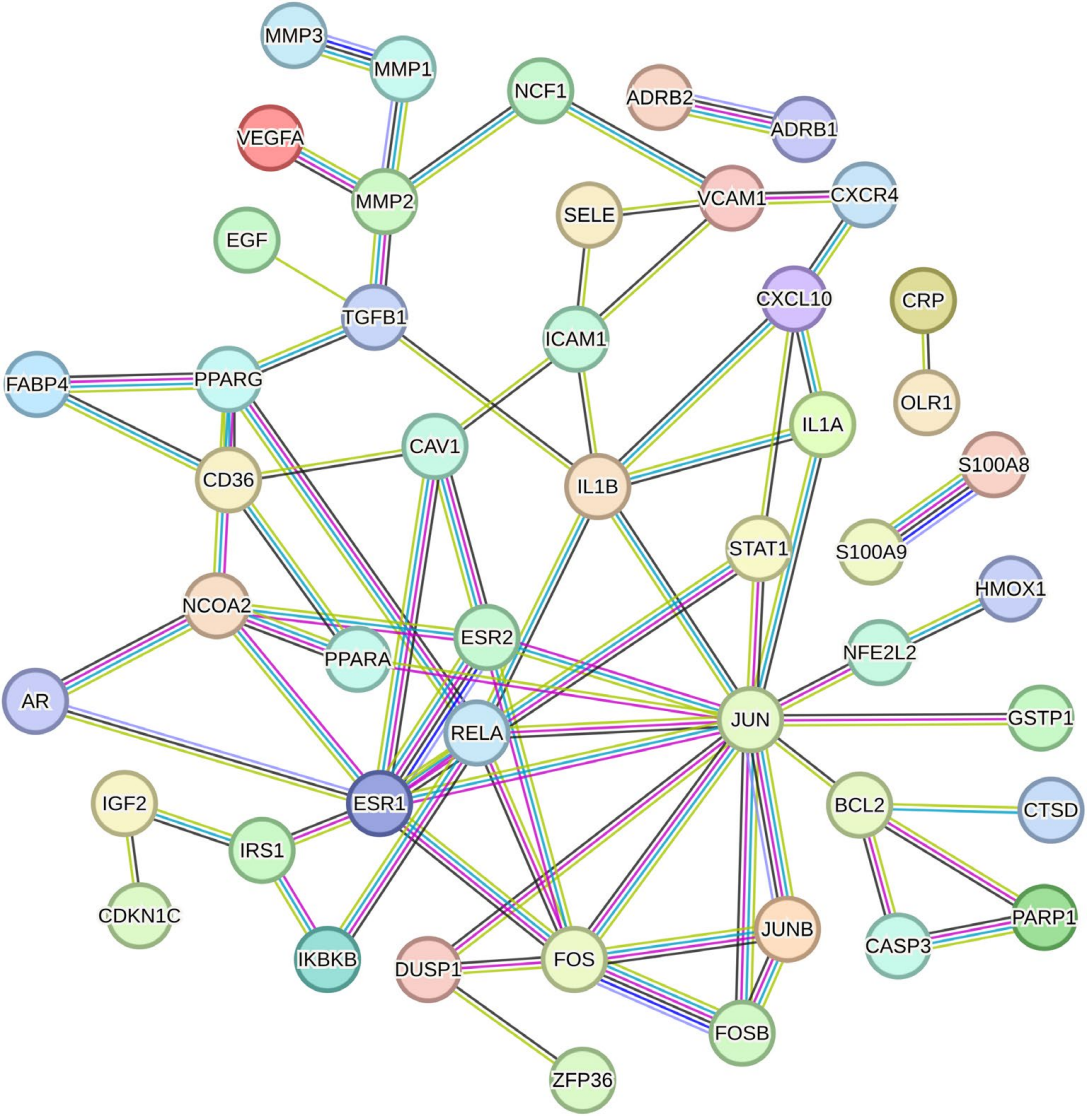
The interaction network between CTPMs and CTs for NK cells in advanced atherosclerosis.

**FIGURE 5:**
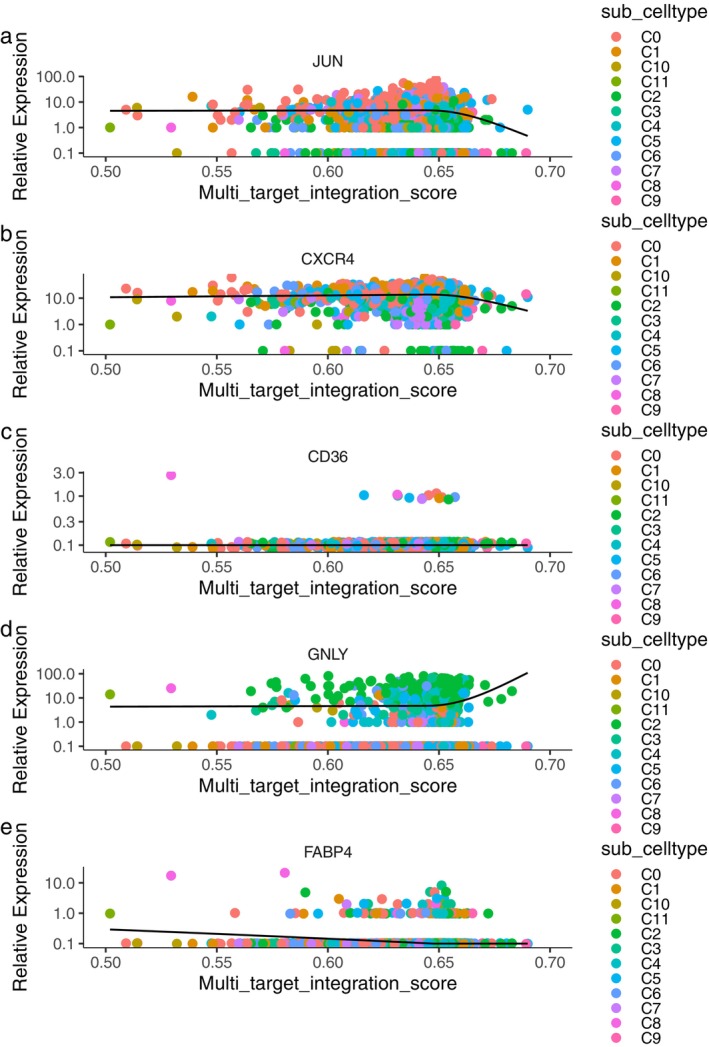
(a) Expressions of JUN in various cell subtypes with MTIS changes. (b) Expressions of CXCR4 in various cell subtypes with MTIS changes. (c) Expressions of CD36 in various cell subtypes with MTIS changes. (d) Expressions of GNLY in various cell subtypes with MTIS changes. (e) Expressions of FABP4 in various cell subtypes with MTIS changes.

**FIGURE 6:**
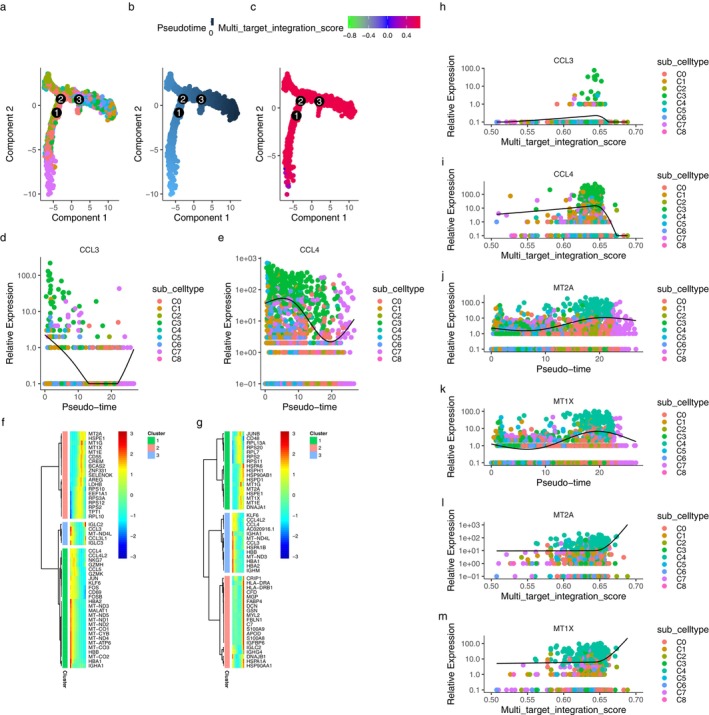
(a) Distributions of T cell subtypes in trajectory analysis. (b) Distributions of Pseudotime in trajectory analysis. (c) Distributions of MTIS in trajectory analysis. (d) Expressions of CCL3 in various cell subtypes with pseudotime changes. (e) Expressions of CCL4 in various cell subtypes with pseudotime changes. (f) Heatmap of the top 50 differentially expressed genes with pseudotime changes. (g) Heatmap of the top 50 differentially expressed genes with MTIS ges. (h) Expressions of CCL3 in various cell subtypes with MTIS changes. (i) Expressions of CCL4 in various cell subtypes with MTIS changes. (j) Expressions of MT2A in various cell subtypes with pseudotime changes. (k) Expressions of MT1X in various cell subtypes with pseudotime changes. (l) Expressions of MT2A in various cell subtypes with MTIS changes. (m) Expressions of MT1X in various cell subtypes with MTIS changes.

Figure 3a–c show the distribution of cell subtypes, pseudo time, and MTIS, respectively, of NK cells in the advanced atherosclerosis group in the trajectory analysis. The numbers in the figure represent branch points, and the cells were clustered and ranked according to pseudo time. Cell subtypes were determined using the FindClusters algorithm. The figure shows that many cell subtypes were distributed at various stages of pseudo time, but subtypes C8 and C10 were the main subtypes at the end of pseudotime. Negative MTIS was mainly concentrated at the end of pseudotime. Figure 3i clearly shows that the MTIS of C10 varied greatly with pseudo time and was mainly negative. Therefore, the C10 subtype of NK cells may be a special cell type involved in the progression of atherosclerosis, without knowing whether KGs are involved. Figure 3d shows the top 50 differentially expressed genes with pseudo time changes (File S8). The pseudo time values in the figure increase from left to right, with red representing high gene expression levels and blue representing low gene expression levels. The differentially expressed genes were grouped into three clusters. The first cluster included the RGCC, FOS, JUN, JUND, JUNB, CCL4, IL7R, and CXCR4 genes. Genes in the first cluster had high expression levels at low pseudo time values and low expression levels at high pseudo time values. FOS, FOSB, JUNB, JUN, and ZFP36 are the AP‐1 transcription factors that respond to various stimuli by regulating gene expression. AP‐1 controls many cellular processes including differentiation, proliferation, and apoptosis. CCL4, IL7R, and CXCR4 are chemokines associated with inflammation. Therefore, the genes in the first cluster are mainly involved in the response to inflammation and transcriptional regulation of immune cells. The genes in the second cluster included PTN, CD36, CAV1, and SPARCL1, which may be involved in the regulation of macrophage uptake of oxidised low‐density lipoproteins. The genes in the third cluster included S100A8, S100A9, GZMH, KLRD1, and FGFBP2, which may be involved in the regulation of calcium‐binding proteins during inflammatory responses. Figure 3h showed the top 50 differentially expressed genes with changes in MTIS (detailed in File S9), where MTIS values increased from left to right. Similarly, it can be inferred that the genes in the first cluster included S100A8, S100A9, MYL2, HLA‐DRB1, HLA‐DRA, CRIP1, FABP4, and CD36. The second cluster included HBB, IGHA2, AREG, and HSPA6. The third cluster included IGKC, ID2, FOS, JUN, and FOSB. MTIS reflected the potential mechanism of hawthorn on the PVAT of atherosclerosis, and pseudo time could reflect important changes in gene expression at each stage of atherosclerosis progression. Therefore, the common genes of both could reflect the important hawthorn genes in PVAT during atherosclerosis progression. A total of 14 common targets between pseudo time‐differential gene analysis and MTIS‐differential gene analysis (CTPMs) were obtained, and the detailed CTPMs in NK cells were JUN, GNLY, GZMK, FABP4, FOS, JUNB, FOSB, S100A9, CXCR4, ZFP36L2, DUSP1, S100A8, CD36, and ZFP36. To identify the key CTPMs, the interaction network between CTPMs and CTs was generated with a parameter of high confidence score in the STRING database (Hamosh et al. 2021) and shown in Figure 4. CTPMs that interact with at least two other CTs were considered key CTPMs. The key CTPMs were JUN, CXCR4, and CD36. In addition, Figure 3b shows the two‐dimensional relationships of differentially expressed genes between −log10 (*q*‐values) of pseudo time and MTIS, with larger values indicating higher significance. The common differentially expressed genes with most significance in pseudoime and MTIS (Co‐Most Significant targets) were GNLY and FABP4. Key mediating genes (KGs) included CTPMs that interacted with at least two CTs, as well as Co‐Most Significant targets. Thus, KGs of NK cells for hawthorn's effects on the PVAT microenvironment of atherosclerosis were JUN, CXCR4, CD36, GNLY, and FABP4. The expression levels of the five genes in each cell subtype with pseudo time changes are shown in (e), (f), (g), (k), and (l) of Figure 3. The expression levels of the five genes in each cell subtype with changes in MTIS are shown in (a), (b), (c), (d), and (e) of Figure 5. From the comparisons of the two groups of figures, it can be seen that in the pseudo time‐variation figure, the expression level of JUN in the C0 subtype was mainly concentrated at the beginning of pseudotime. In the MTIS‐variation figure, the expression level of JUN in the C0 subtype was mainly concentrated in the 0.6 to 0.65 region of MTIS. The C0 subtype exhibited specific variability for involving KGs in both groups of figures. Similarly, the C11 subtype also exhibited specific variability in the involvement of KGs in the expression level of CNLY in both groups of figures. We determined that the most important NK cell subtypes were C0 and C11 for the effect of hawthorn on advanced atherosclerosis (see Figure 6).

#### 3.3.2 Analysis of T Cells in Advanced Atherosclerosis

**FIGURE 7:**
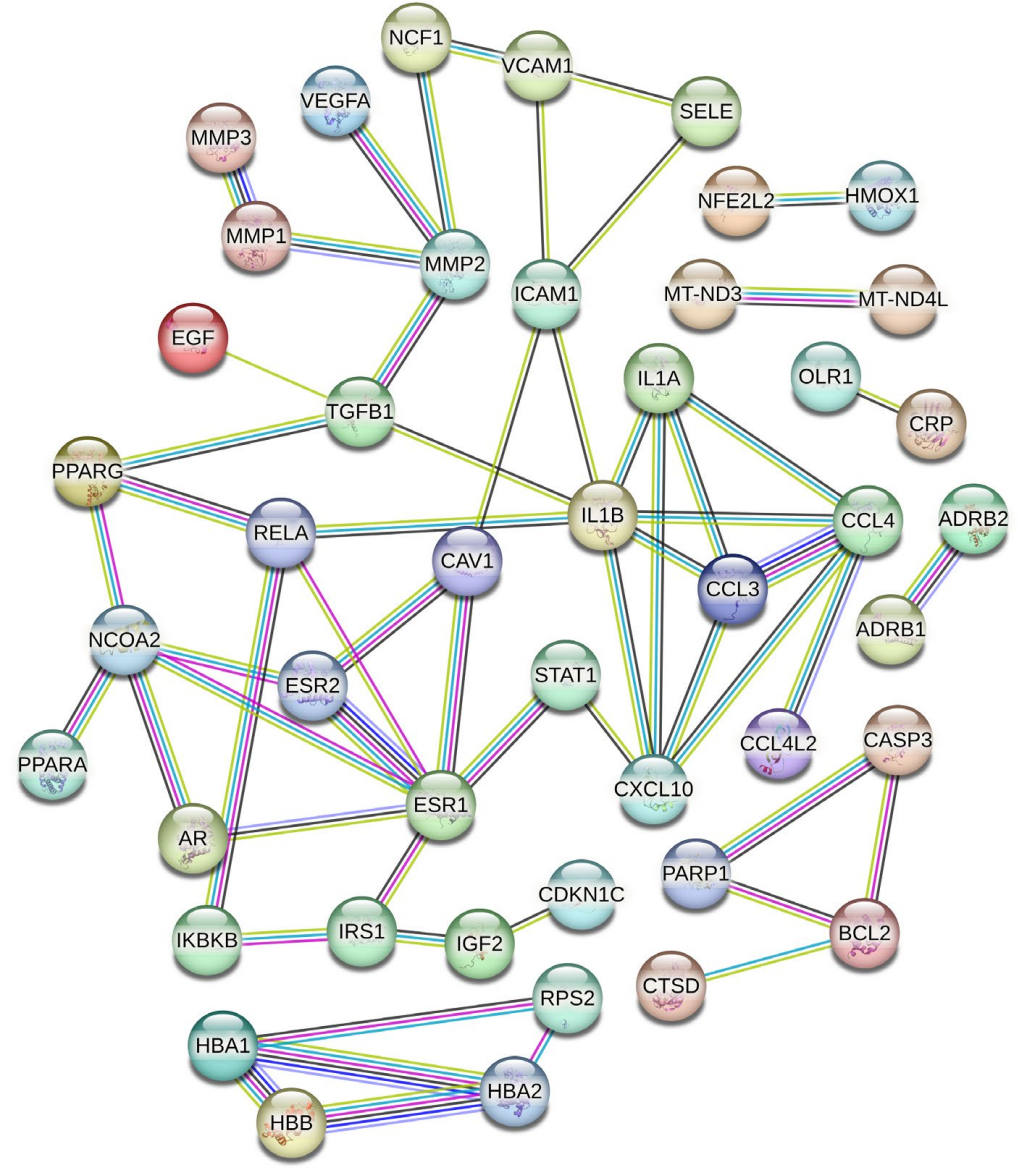
The interaction network between CTPMs and CTs for T cells in advanced atherosclerosis.

Using a similar analysis method as described above, C7 might be a key subtype between pseudo‐time and MTIS, without knowing whether KGs were involved, according to (a), (b), and (c) of Figure 7. Figure 7f shows the top 50 pseudo‐time‐differential genes of T cells in the advanced stage of atherosclerosis (detailed in File S10), which were divided into three clusters. The first cluster mainly included metallothionein‐related genes, such as MT2A and MT1G, ribosome‐related genes, RPS, and CD55. The second cluster included the inflammatory chemokine CCL3. The third cluster included CCL4, CCL5, JUN, FOS, CD69, and MT‐related genes. Figure 7g showed the top 50 MTIS‐differentially expressed genes of T cells in advanced atherosclerosis (File S11). The changes in gene expression levels in the third cluster were relatively consistent, showing that the gene expression levels were low when MTIS was low and the gene expression levels were high when MTIS was high, including CRIP1, HLA‐DRA, CFD, MGP, and FABP4. The CTPMs in T cells were HBB, HBA2, HBA1, MT2A, MT1X, CCL3, CCL4, CCL4L2, MT‐ND3, MT1E, MT1G, IGLC2, IGHA1, KLF6, MT‐ND4L, HSPE1, and RPS2. The interaction network between CTPMs and CTs for T cells in advanced atherosclerosis is shown in Figure 7. CTPMs that interacted with at least two CTs were CCL3 and CCL4. The Co‐Most significant targets were MT2A and MT1X in Figure 7f,g. The KGs of T cells involved in the effects of hawthorn on the PVAT microenvironment in atherosclerosis were CCL3, CCL4, MT2A, and MT1X. Comparing Figure 7d,h, as well as Figure 7e,i, we determined that C3 subtype exhibited specific variability in the involvement of KGs in both pseudo‐time and MTIS. From the comparison of (j), (k), (l), and (m) in Figure 7, it can be concluded that the C4 subtype exhibited specific variability in the involvement of KGs in both pseudo‐time and MTIS. Therefore, the important subtypes of T cells were C3 and C4 for the effect of hawthorn on advanced atherosclerosis.

#### 3.3.3 Analysis of Monocyte in the Early Stage of Atherosclerosis

**FIGURE 8:**
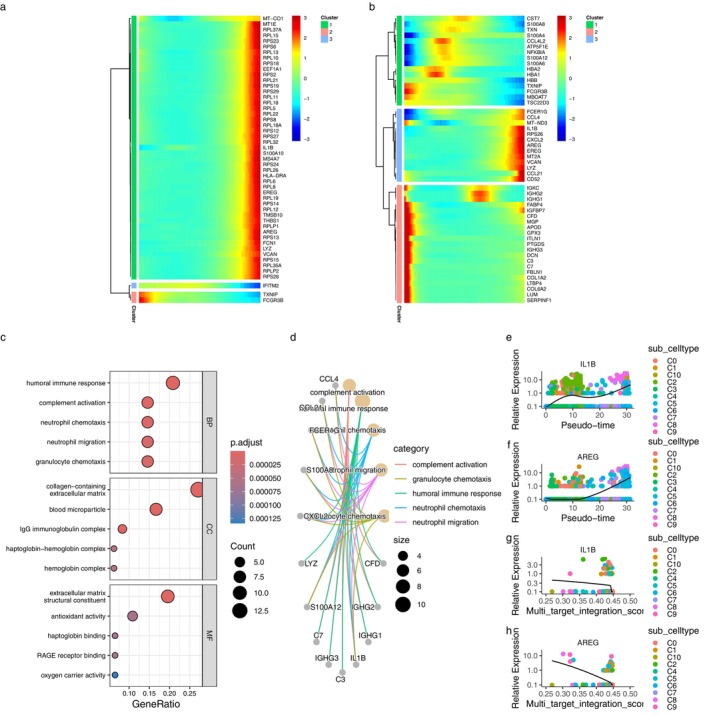
(a) Heatmap of the top 50 differentially expressed genes with pseudotime changes. (b) Heatmap of the top 50 differentially expressed genes with MTIS changes. (c) Enrichment analysis of MTIS differentially expressed genes. (d) The associations between the top 5 pathways of enrichment analysis of MTIS differentially expressed genes and the involved genes. (e) Expressions of IL1B in various cell subtypes with pseudotime changes. (f) Expressions of AREG in various cell subtypes with pseudotime changes. (g) Expressions of IL1B in various cell subtypes with MTIS changes. (h) Expressions of AREG in various cell subtypes with MTIS changes.

**FIGURE 9:**
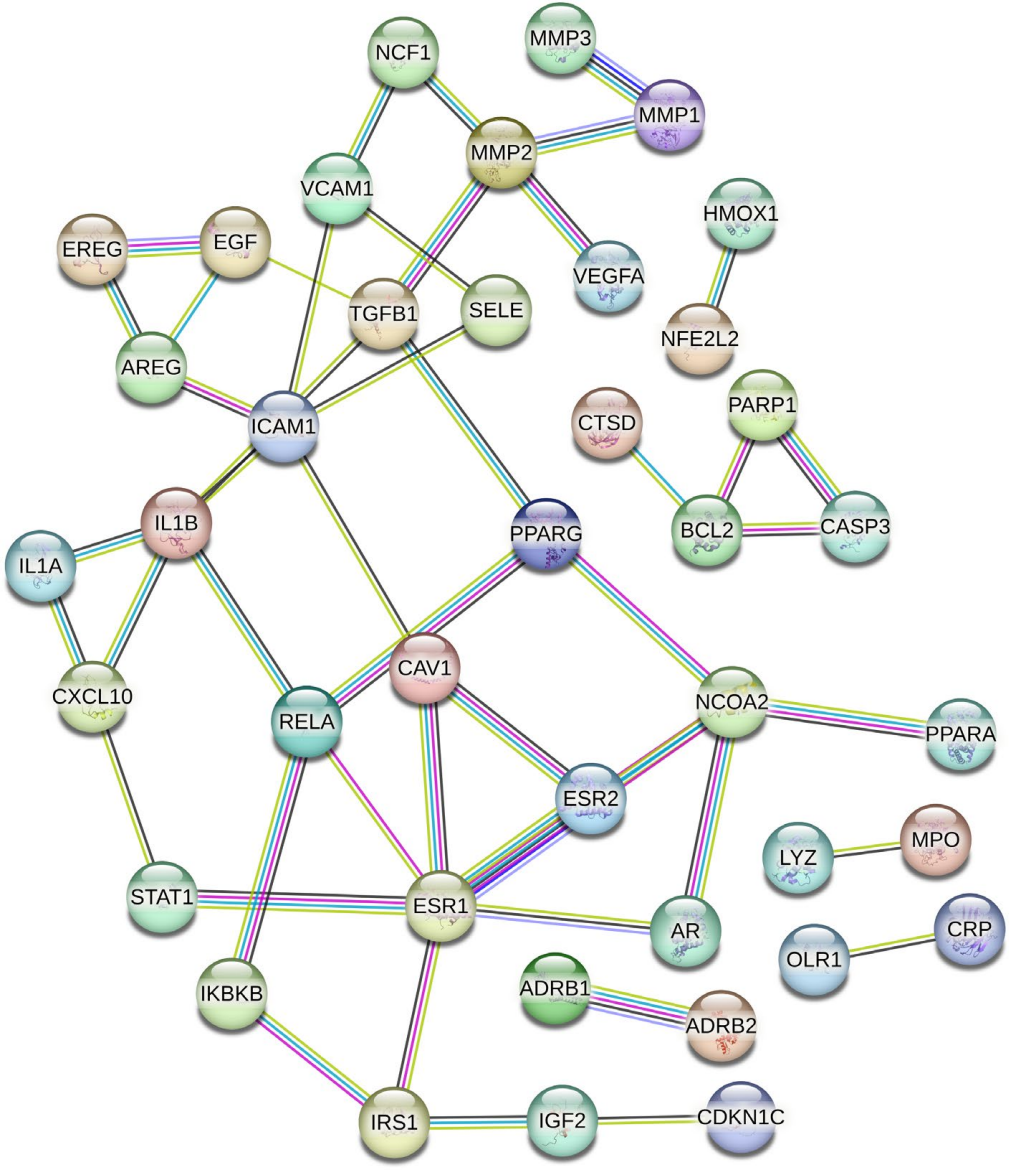
The interaction network between CTPMs and CTs for monocyte in early stage of atherosclerosis.

As shown in (a) of Figure 8, most of the pseudo‐time‐differential genes (detailed in File S2) were clustered into one cluster composed of ribosome‐related genes, such as RPS and RPL. In (b) of Figure 8, the second cluster of MTIS differentially expressed genes (detailed in File S3) mainly included inflammation‐related factor genes such as CCL, IL1B, and CXCL2. The third cluster mainly included IGHC, C3, C7, FABP4, and APOD, which are related to complement immunity or fat synthesis. Figure 8c,d,f show the major pathways for the enrichment analysis of MTIS‐differentially expressed genes (detailed in File S4), as well as their associations with the involved genes. The pathways mainly focused on the complement response, humoral immune response, neutrophil chemotaxis, and migration. It can be inferred that when hawthorn acts on the PVAT of atherosclerosis, there is close communication between monocytes, B cells, and neutrophils. The CTPMs shown in Figure (a) and (b) were TXNIP, FCGR3, FCGR3B, EREG, AREG, VCAN, LYZ, and IL1B. The interaction network between CTPMs and CTs for monocytes in the early stage of atherosclerosis is shown in Figure 9, where IL1B is a common target between CTPMs and CTs, and AREG is the CTPMs that connects with two or more CTs. However, there were no co‐most significant targets between Figure (a) and (b). Therefore, the KGs in the early stages of atherosclerosis are IL1B and AREG. Comparing (e), (f), (g), and (h) in Figure 8, no important cell subtypes for monocytes in the early stages of atherosclerosis were found.

### 3.4 Immune Infiltration Landscape Analysis of Key Cell Subtypes

**FIGURE 10:**
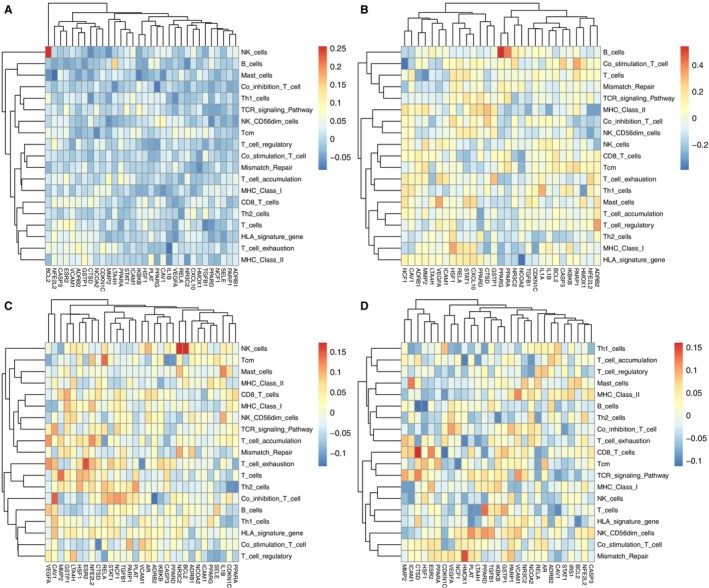
(a) The immune infiltration analysis of C0 in NK cells. (b) The immune infiltration analysis of C11 in NK cells. (c) The immune infiltration analysis of C3 in T cells. (d) The immune infiltration analysis of C4 in T cells.

Based on the above analysis, the C0 and C11 subtypes of NK cells and the C3 and C4 subtypes of T cells are important subtypes of KGs. The immune infiltration landscape analysis of the four subtypes is shown in Figure 10 (detailed in Files S15–S18). The horizontal axis represents CTs, and the vertical axis represents the immune infiltration term. Most CTs in Figure 10a show a negative correlation with immune infiltration, indicating that CTs might be involved in immune suppression in the C0 subtype of NK cells. Figure 10a shows that BCL2 was significantly positively correlated with NK cells compared to other grid colours in the background of Figure 10a. Similarly, we obtained CTs with a significantly positive correlation to the immune infiltration term and a significantly negative correlation with the immune infiltration term in all graphs. CTs were positively correlated with immune infiltration, including BCL2, PPARG, NR3C2, CTSD, and HMOX1. PPARG was positively correlated with B cells, NR3C2 was positively correlated with B and NK cells, CTSD was positively correlated with CD8+T cells, and HMOX1 was positively correlated with mismatch repair. CTs that negatively correlated with immune infiltration included IL1B, PPARG, NFE2L2, NCF1, NCOA2, ADRB2, HSF1, CTSD, CDKN1C, and HMOX1. IL1B was negatively correlated with T cell exhaustion, PPARG was negatively correlated with the co‐inhibition T cells, NFE2L2 was negatively correlated with mast cells, and NCF1 was negatively correlated with the co‐stimulation T cells. NCOA2 expression negatively correlated with HLA signatures. ADRB2 was negatively correlated with co‐inhibition T cells. HSF1 expression was negatively correlated with B cells. CTSD was negatively correlated with B cells and co‐stimulated T cells. CDKN1C expression was negatively correlated with CD8+T cells. HMOX1 was negatively correlated with T cell exhaustion and accumulation. Targets with coefficients most correlated with the immune microenvironment signatures in multiple immune infiltration landscape maps were considered key targets related to immunity (KTRI). KTRI for hawthorn in the PVAT microenvironment of atherosclerosis were CTSD, PPARG, and Hmox1.

**FIGURE 11:**
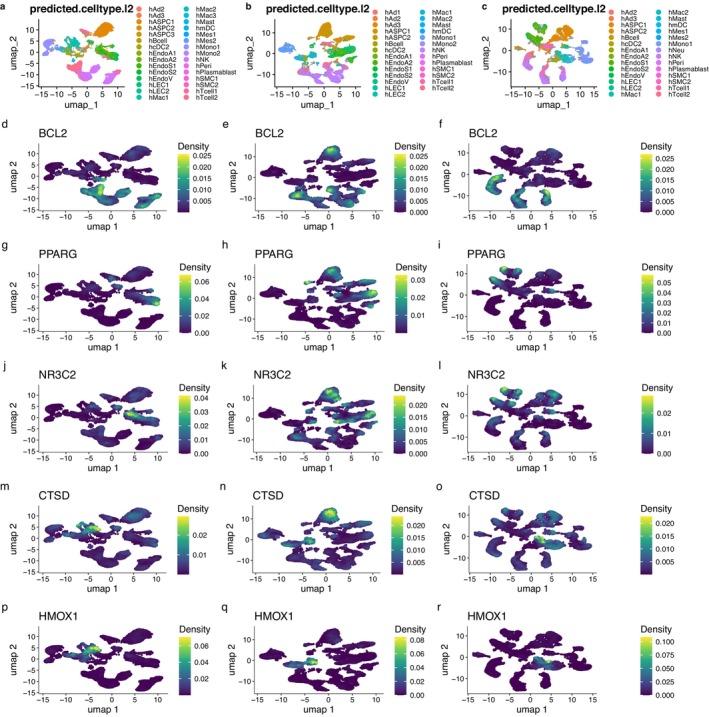
(a) Distributions of cell types in cell clusters in early stage atherosclerosis group. (b) Distributions of cell types in cell clusters in advanced atherosclerosis group. (c) Distributions of cell types in cell clusters in non‐atherosclerosis group. (d) to (f) Density distributions of BCL2 in early stage atherosclerosis group, advanced atherosclerosis group, and non‐atherosclerosis group, respectively. (g) to (i) Density distributions of PPARG in early stage atherosclerosis group, advanced atherosclerosis group, and non‐atherosclerosis group, respectively. (j) to (l) Density distributions of NR3C2 in early stage atherosclerosis group, advanced atherosclerosis group, and non‐atherosclerosis group, respectively. (m) to (o) Density distributions of CTSD in early stage atherosclerosis group, advanced atherosclerosis group, and non‐atherosclerosis group, respectively. (p) to (r) Density distributions of HMOX1 in early stage atherosclerosis group, advanced atherosclerosis group and non‐atherosclerosis group, respectively.

**FIGURE 12:**
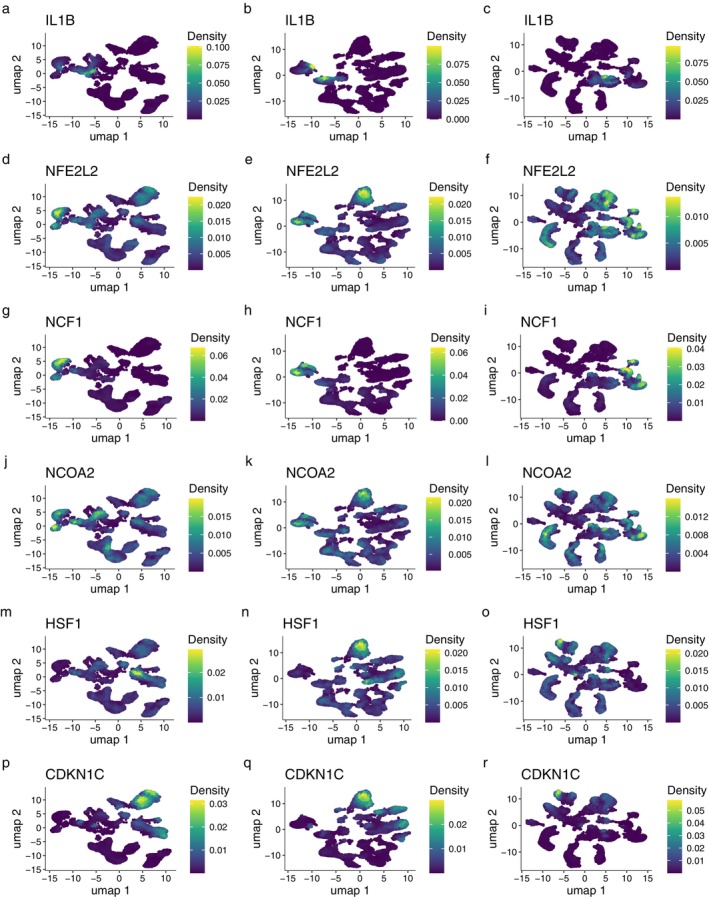
(a) to (c) Density distributions of IL1B in early stage atherosclerosis group, advanced atherosclerosis group, and non‐atherosclerosis group, respectively. (d) to (f) Density distributions of NFE2L2 in early stage atherosclerosis group, advanced atherosclerosis group, and non‐atherosclerosis group, respectively. (g) to (i) Density distributions of NCF1 in early stage atherosclerosis group, advanced atherosclerosis group, and non‐atherosclerosis group, respectively. (j) to (l) Density distributions of NCOA2 in early stage atherosclerosis group, advanced atherosclerosis group, and non‐atherosclerosis group, respectively. (m) to (o) Density distributions of HSF1 in early stage atherosclerosis group, advanced atherosclerosis group, and non‐atherosclerosis group, respectively. (p) to (r) Density distributions of CDKN1C in early stage atherosclerosis group, advanced atherosclerosis group, and non‐atherosclerosis group, respectively.

Figures 11 and 12 showed the density distributions of the major CTs, with a significant correlation with the immune infiltration term. We focused on the density distributions of KTRI between the early‐stage atherosclerosis, advanced atherosclerosis, and non‐atherosclerosis groups. Comparing (a) and (m) of Figure 11, we can see that in the early‐stage atherosclerosis group, the high expression region of CTSD was mainly distributed in MAC cells. Compared with (b) and (n) of Figure 11, in the advanced atherosclerosis group, the high expression region of CTSD was mainly distributed in ASPC cells. In the non‐atherosclerotic group, the high‐expression region of CTSD was mainly distributed in the MAC cells. Further analysis showed that in the early‐stage atherosclerotic and non‐atherosclerotic groups, the high‐expression region of PPARG was mainly distributed in endothelial cells, whereas in the advanced atherosclerotic group, the high‐expression region of PPARG was mainly distributed in ASPC cells. In the comparison of the three groups, the high‐expression regions of HMOX1 were all located in MAC cells. Furthermore, in Figures 11 and 12, compared with the non‐atherosclerotic group, most other genes were highlighted in the regions of ASPC in the advanced atherosclerotic group. The results showed that, except for MAC cells (macrophages), ASPC were important non‐immune cells related to the microenvironment of hawthorn on the PVAT of atherosclerosis.

### 3.5 Cell Communication Analysis of Non‐Immune Cells in the MTIS‐Differential Cell Types

**FIGURE 13:**
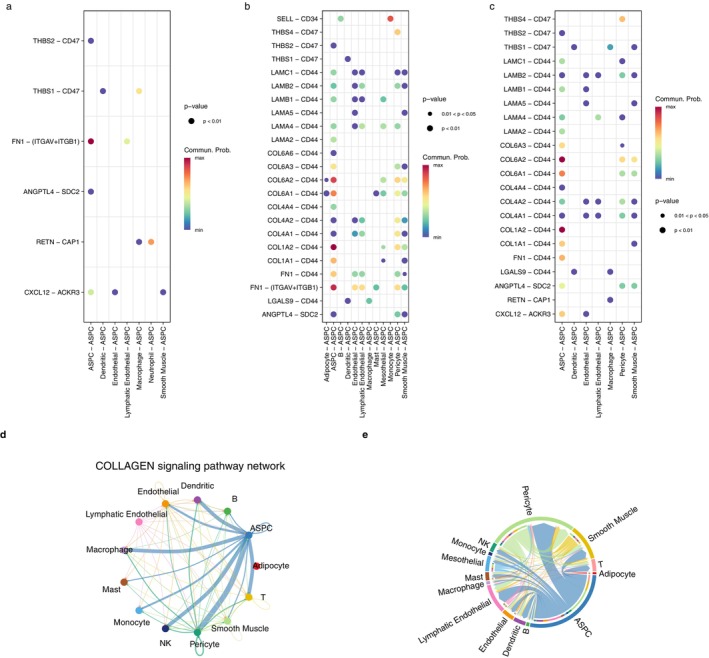
(a) Pathways and ligand‐receptor pairs of communications between other cells and ASPC in the non‐atherosclerotic group, CTs involved in the pathways and ligand‐receptor pairs or CTs interacted with the pathways and ligand‐receptor pairs. (b) Pathways and ligand‐receptor pairs of communications between other cells and ASPC in the early stage atherosclerotic group, CTs involved in the pathways and ligand‐receptor pairs or CTs interacted with the pathways and ligand‐receptor pairs. (c) Pathways and ligand‐receptor pairs of communications between other cells and ASPC in the advanced atherosclerotic group, CTs involved in the pathways and ligand‐receptor pairs or CTs interacted with the pathways and ligand‐receptor pairs. (d) Communication strengths between ASPC and other cells on the COLLAGEN pathway in the advanced atherosclerotic group. (e) Communication strengths between ASPC and other cells on the COLLAGEN pathway in the early stage atherosclerotic group.

**FIGURE 14:**
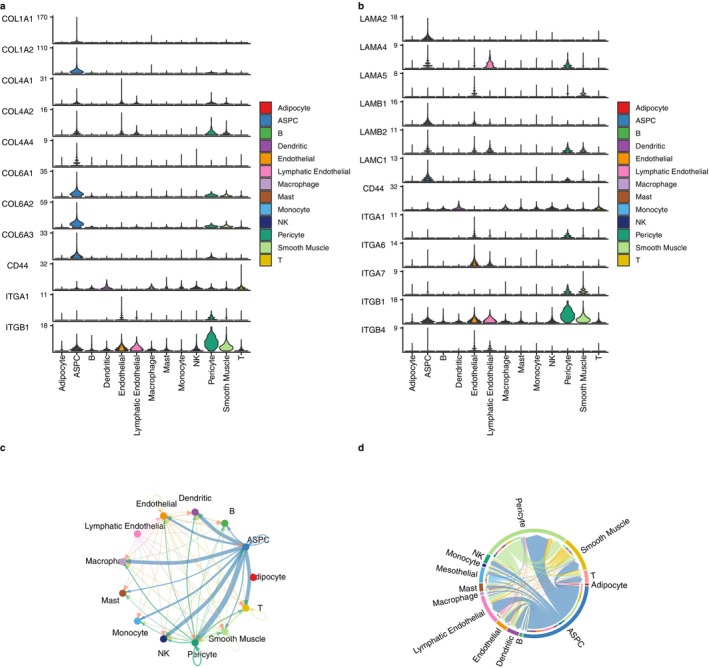
(a) The expression levels of genes involved in the COLLAGEN pathway in various cells in the advanced atherosclerotic group. (b) The expression levels of genes involved in the LAMININ pathway in various cells in the advanced atherosclerotic group. (c) Communication strengths between ASPC and other cells on the LAMININ pathway in the advanced atherosclerotic group. (d) Communication strengths between ASPC and other cells on the LAMININ pathway in the early stage atherosclerotic group.

ASPC are non‐immune cells in MTIS‐differential cell types (NICIM), and they are also important non‐immune cells related to the microenvironment of hawthorn on PVAT of atherosclerosis. Analysis of cell communication between ASPC and other cells was performed. Figure 13a–c show pathways and ligand‐receptor pairs of communication between other cells and ASPC in the non‐atherosclerotic, early stage atherosclerotic, and advanced atherosclerotic groups, respectively. CTs are involved in these pathways and ligand‐receptor pairs or CTs interact with these pathways and ligand‐receptor pairs. Compared to the non‐atherosclerotic group, the early‐stage atherosclerotic and advanced atherosclerotic groups mainly showed increased COLLAGEN and LAMININ pathways, and the increased ligand‐receptor pairs included LAMA‐CD44, LAMB‐CD44, LAMC‐CD44, COL6A‐CD44, and FN1‐CD44. The highest communication probability (red dot) focused on the communication of the ASPC itself as well as the communication between monocytes and ASPC. As shown in Figure 13d,e, in the early stage of atherosclerosis, the cells with high communication strength with the ASPC included pericytes, smooth muscle cells, and lymphatic endothelial cells. In advanced atherosclerosis, cells with high communication strength with ASPC included pericytes, macrophages, NK cells, and T cells. This indicates that immune cell infiltration increases during advanced atherosclerosis. Figure 14a reveals that the major genes of the COLLAGEN pathway are most expressed in ASPC cells, while Figure 14b reveals that the major genes of the LAMININ pathway are highly expressed in ASPC cells and are also expressed in endothelial cells and pericytes. In (c) and (d) of Figure 14, the communication strengths between the ASPC and other cells on the LAMININ pathway in the advanced atherosclerotic and early‐stage atherosclerotic groups are similar to the COLLAGEN pathway in (d) and (e) of Figure 13, respectively. In short, the regulatory mechanism was related to the COLLAGEN pathway and LAMININ pathways, and immune infiltration increased in advanced atherosclerosis. These two pathways involve the regulation of collagen and laminin, which indicates that the effect of hawthorn on PVAT in atherosclerosis might involve plaque fibrosis or the repair process of the damaged endothelium.

### 3.6 Model Evaluation

**FIGURE 15:**
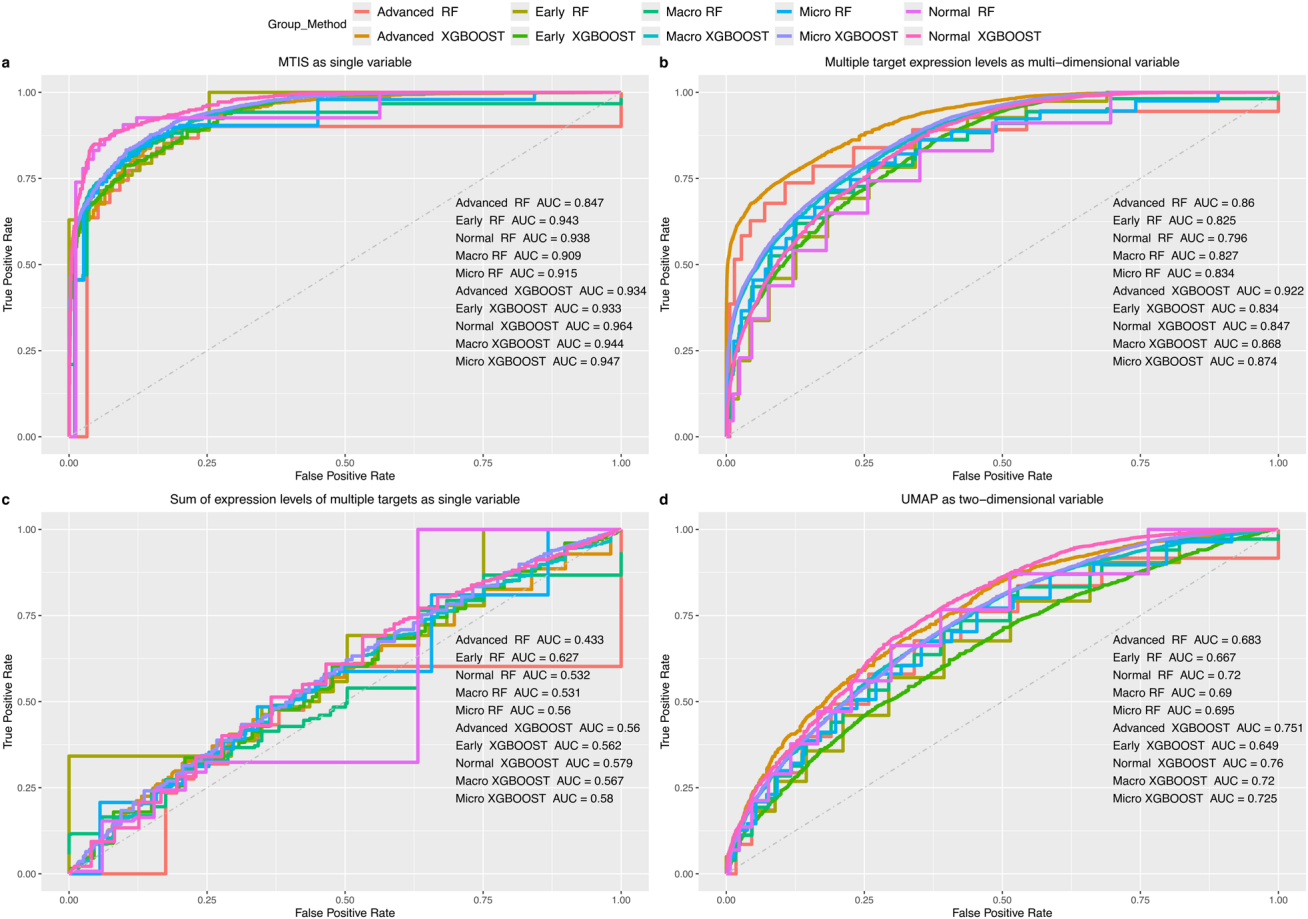
(a) ROC curves and AUC values in different groups when MTIS was used as single‐dimensional input variable. (b) ROC curves and AUC values in different groups when the expression levels of multiple genes in each cell were used as multi‐dimensional input variables. (c) ROC curves and AUC values in different groups when the sum of the expression levels of multiple genes in each cell was used as single‐dimensional input variable. (d) ROC curves and AUC values in different groups when the UMAP‐generated two‐dimensional variable of each cell was used as a two‐dimensional input variable.

As shown in Figure 15, compared with the other baseline models, the ROC curves and AUC values were clearly in a leading position when MTIS was used as a single‐dimensional input variable. In Figure 15a, all ROC curves are significantly closer to the upper left corner of the coordinates, which indicates that using the MTIS of cells to predict the group to which the cells belonged had higher performance. That is to say, MTIS could more accurately distinguish the pathological changes of different groups (advanced atherosclerosis, early‐stage atherosclerosis, and non‐atherosclerosis group); the three groups are marked as “Advanced”, “Early” and “Normal” in Figure 15, respectively. Micro and macro represent the micro average and the macro average of the algorithm's predictive performance, respectively, in the three groups mentioned above. When the RF algorithm was used, the AUC values of the micro‐average and macro‐average groups were 0.915 and 0.909, respectively. When the XGBSOOT algorithm was used, the AUC values of the micro‐average and macro‐average groups were 0.947 and 0.944, respectively. For the RF and XGTBOOST algorithms, the micro‐average and macro‐average groups in Figure 15a had the highest values among the four test models. In addition, as shown in Figure 15a, for the prediction performance in a specific group, almost all groups had the highest prediction performance among the four test models. Therefore, MTIS could accurately reflect the comprehensive (integrated) state of expression levels of multiple genes in cells in different groups, and the deep learning auto‐encoding neural network was a model with excellent performance.

Figure 15b shows the second‐ranked score, where the expression levels of multiple genes in each cell were used as multidimensional input variables. The original expression levels of multiple genes were multidimensional with the richest information content; however, it was still difficult to surpass MTIS in this situation. Figure 15c has the worst score, indicating that even when converted to one‐dimensional variables, such as MTIS, the integration process of the expression data of multiple genes was not a simple process, such as summation, which often did not achieve good results. Figure 15d shows the predictive performance of two‐dimensional variables generated by the UMAP algorithm based on the expression levels of multiple genes. In the field of single‐cell data processing, the UMAP and t‐SNE algorithms are well‐known dimensional‐reduction algorithms that can convert multidimensional data of the expression levels of multiple genes in a single cell into two dimensions for further cell visualisation or data analysis. In this study, the UMAP algorithm ranked third, indicating that MTIS had sufficient dimensional reduction advantages and could accurately integrate (encoded) the expression levels of multiple genes into one‐dimensional variable values.

### 3.7 Experiment Validation

**FIGURE 16:**
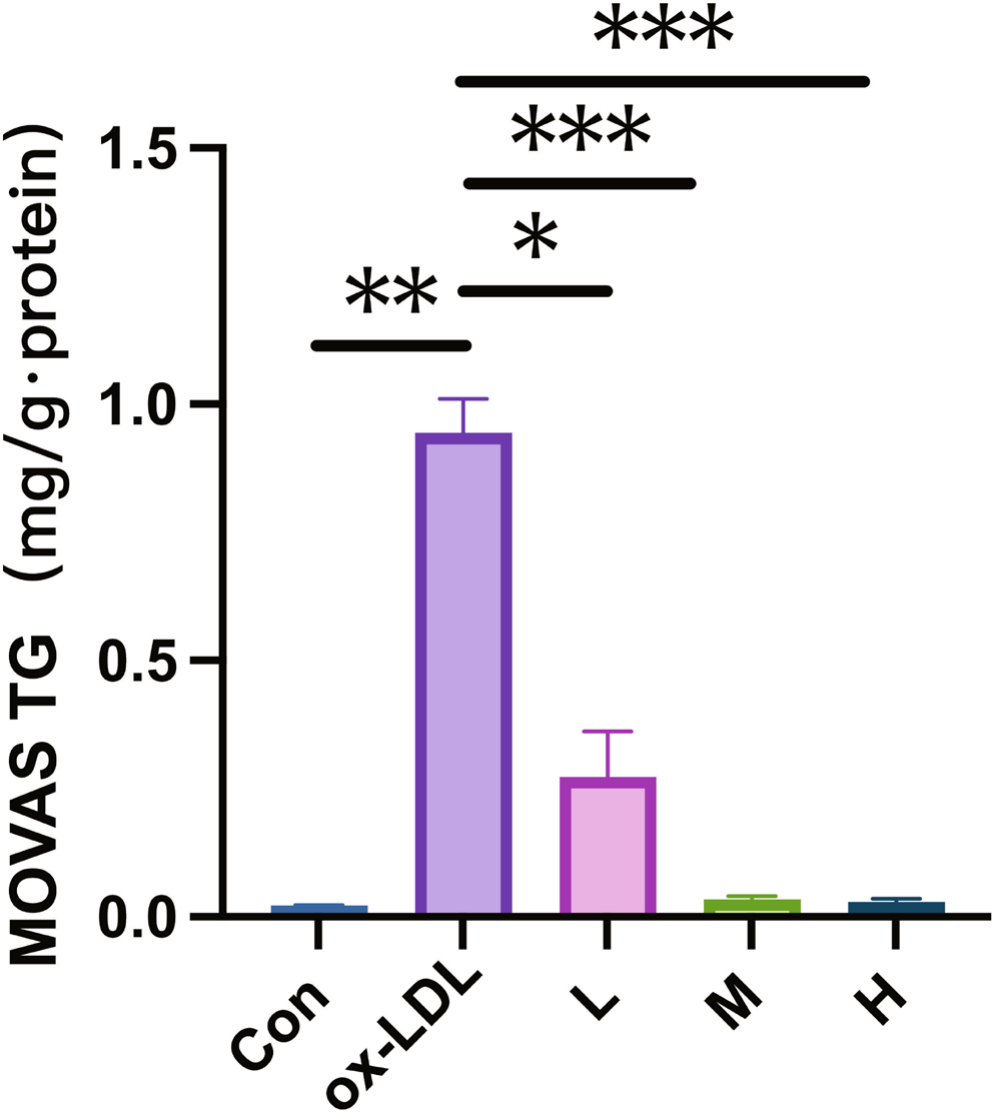
TG expression in co‐culture of MOVAS and 3T3‐L1 cells group and control group. “Con” represents the control group, “ox‐LDL” represents the model group, and “H”, “M”, and “L” represent the three experimental groups, each of which was intervened with different concentrations of hawthorn extract (high, medium, low) on the basis of the model group. *p‐value less than 0.05. **p‐value less than 0.01. ***p‐value less than 0.001.

A comparative analysis of TG expression in a co‐culture of MOVAS and 3T3‐L1 cells from various experimental groups was conducted. A significant increase in TG expression was observed in the model group compared to that in the control group, as illustrated in Figure 16. In the hawthorn‐treated groups, TG levels were notably lower than those in the model group (*p* < 0.01), with the high‐ and medium‐dose groups exhibiting lower TG levels than the low‐dose group. These findings confirmed the successful establishment of the co‐culture of MOVAS and 3T3‐L1 cells model under all experimental conditions.

**FIGURE 17:**
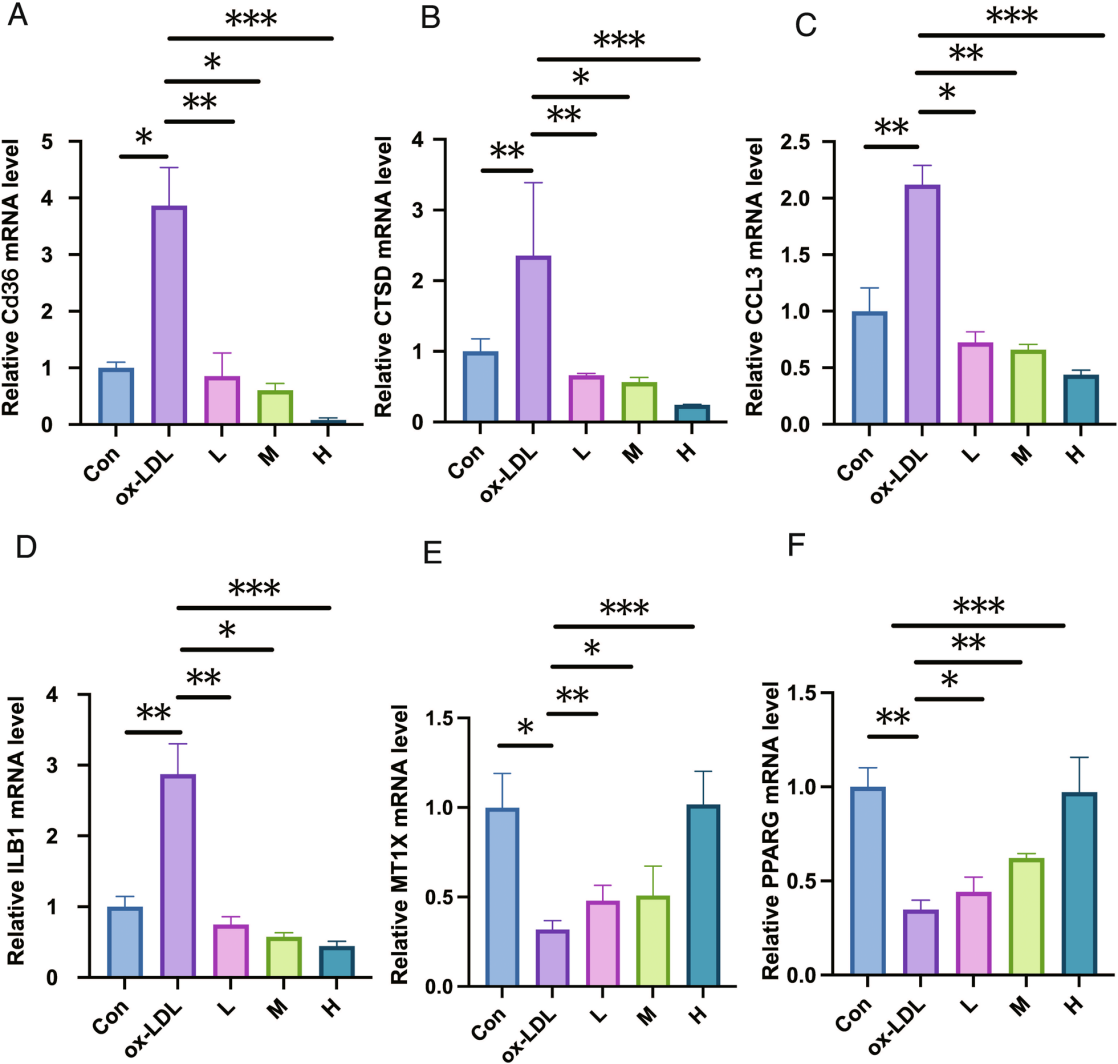
Representative qRT‐PCR analysis of CD36, CTSD, CCL3, IL1B, PPARG, and MT1X expression in co‐culture of MOVAS and 3T3‐L1 cells. “Con” represents the control group, “ox‐LDL” represents the model group, and “H”, “M”, and “L” represent the three experimental groups, each of which was intervened with different concentrations of hawthorn extract (high, medium, low) on the basis of the model group. *p‐value less than 0.05. **p‐value less than 0.01. ***p‐value less than 0.001.

In addition, qRT‐PCR analysis of target gene expression across different cell groups revealed notable differences. Specifically, compared with the control group, the expression levels of CD36, CTSD, CCL3, and IL1B were significantly elevated in the model group, while the expression levels of PPARG and MT1X were decreased in the model group, as shown in Figure 17. In contrast, the groups treated (intervened) with hawthorn extract exhibited a marked decrease in target expression compared to the model group (*p* < 0.01). CD36, CTSD, CCL3, and IL1B expression decreased in a dose‐dependent manner, whereas PPARG and MT1X expression increased in a dose‐dependent manner. This reveals that PPARG and MT1X may be beneficial genes for atherosclerosis. These findings suggest that CD36, CTSD, CCL3, IL1B, PPARG, and MT1X are potential targets for the action of hawthorn in the PVAT microenvironment of atherosclerosis and are consistent with the following discussion.

## 4 Discussion

### 4.1 The Innovation, Advantages, and Disadvantages of the Developed New Research Method Based on Artificial Intelligence in This Study

We proposed a novel research method for revealing the quantitative multi‐target integrative effects of plants on artificial intelligence and applied it to analyse the potential mechanism of hawthorn in the PVAT microenvironment of atherosclerosis. Compared with existing bioinformatics analysis methods, the advantage of this method is that it utilises artificial intelligence to integrate the expression levels of multiple targets of plants (hawthorn) in the single‐cell transcriptome into encoded values called Multi Target Integration Score (MTIS), and generates different MTIS for each cell to reveal the landscape and quantitative mapping of multi‐target potential effects of plants on disease at the single‐cell level. Because MTIS comprehensively reflects the expression levels of common targets of plants in a single‐cell transcriptome, it could reflect the differences in expression levels of common targets of plants in different transcriptomes by comparing the differences in MTIS between the non‐atherosclerosis and atherosclerosis groups. Therefore, the potential mechanisms of action of plants on diseases can be explored using MTIS. In addition, we used MTIS to compare the potential effects of hawthorn on various cell types to further identify the major effector cells, genes, or pathways involved in the potential mechanism of action of plants. Furthermore, we used a new analytical technique, the MTIS pseudo‐time analysis, to determine the potential mechanism. These methods are not available in the current literature and represent the innovation and advantages of this study. Of course, there were also some limitations to this study, such as the need to construct and train deep neural network models, which are more complex than existing analysis methods. In addition, the method proposed in this study is applicable to multi‐target research, and landscape and quantitative analyses of multi‐target potential integrated effects of plants on diseases at the single‐cell level were achieved. If a drug or food has only a single target for a disease, rather than multiple targets, the method proposed in this study might not be applicable. However, many diseased plants have multiple chemical compositions and targets. Therefore, the novel method proposed in this study has a wide range of applications. Many plants that affect disease are referred to as medicinal plants. For example, ginseng is believed to have the ability to enhance immunity and strengthen the body and has good therapeutic effects on diseases such as fatigue, neurasthenia, low memory, and sub‐health. Using the model and analytical framework of this study, it was possible to analyse which cells were mainly affected by ginseng and which key targets and pathways were involved in its specific mechanism. Another example is Salvia miltiorrhiza Bunge, a well‐known cardiovascular medical plant, which is believed to promote blood circulation and remove blood stasis, and has a good effect on coronary heart disease and arrhythmia. Using the model and analytical framework of this study, it was possible to analyse which specific vascular and myocardial cells are effector cells, which targets could improve the hypoxia tolerance of myocardial cells, and further, which targets could regulate platelet aggregation to prevent thrombosis. Because plants have many different parts such as leaves, fruits, and roots, these parts might also have different effects on the same disease. The model and analytical framework used in this study could also be used for the comparison and quantitative analysis of the effects of different plant parts on the same disease. A potential limitation was that the method used in this study could only analyse potential mechanisms and requires further experimental validation. However, the method used in this study provided specific mechanistic research directions for experimental verification.

### 4.2 The Potential Mechanism of Hawthorn on the Perivascular Adipose Tissue of Atherosclerosis

According to the above analysis, the KGs that affect the PVAT microenvironment in atherosclerosis are JUN, CXCR4, CD36, GNLY, FABP4, CCL3, CCL4, MT2A, MT1X, IL1B, and AREG. KTRI for hawthorns in the PVAT microenvironment of atherosclerosis were CTSD, PPARG, and HMOX1. This regulatory mechanism is also involved in the COLLAGEN pathway and LAMININ pathways mediated by CD44, and immune infiltration increases in advanced atherosclerosis. Under normal physiological conditions, PVAT secretes fat factors with anti‐atherosclerotic effects, such as adiponectin and vaspin. For example, adiponectin had the ability to inhibit inflammatory responses, inhibit the formation of atherosclerosis plaques, and reduce the expressions of adhesion molecules in endothelial cells (Garbuzova et al. 2024; Gianopoulos et al. 2024). Under pathological conditions, PVAT abnormally secretes cytokines such as the pro‐inflammatory cytokine IL‐6 (Simplicio et al. 2023). The lack of a connective tissue barrier between the PVAT and adjacent arteries allows cytokines and fatty acids to overflow into the adventitia, further promoting arterial inflammation or directly affecting lipid deposition and plaque formation (Horimatsu et al. 2018; Stanek et al. 2021; Nosalski and Guzik 2017; Ahmadieh et al. 2020). Therefore, how hawthorn inhibits the secretion of PVAT‐related cytokines or PVAT‐related fatty acids and prevents these harmful factors from migrating and adhering to the adventitia and intima of blood vessels, as well as playing a role in atherosclerosis, is the key mechanism underlying atherosclerosis in the PVAT environment.

As hawthorn has multiple chemical components and targets, its potential mechanism involves multiple aspects. According to the results of this study, the main potential mechanisms of hawthorn on PVAT in atherosclerosis include the following.

1. Regulating the formations of foam cells by targeting CD36, FABP4, and CTSD Macrophages engulf oxidised LDL (Ox‐LDL) to form foam cells, which are deposited in the intima to form plaques, the core mechanism of atherosclerosis (Duan et al. 2024). CD36 is a scavenger receptor (SRs) that recognises Ox‐LDL on the surface of macrophages. It had been shown that in most cases, the uptake of Ox‐LDL by macrophages was mediated by CD36, which was an important mechanism of atherosclerosis (Duan et al. 2024). FABP4 is a cross‐target gene involved in inflammation and lipid metabolism. It is widely involved in inflammatory reactions and lipid metabolism, and is a key gene that promotes atherosclerosis. Studies have shown that the cholesterol content of macrophages in FABP4−/− mice is significantly reduced, and the inflammatory cytokine activity in FABP4−/− mice is weakened (Chen et al. 2022). Therefore, FABP4 could affect the formation of foam cells and the accumulation of lipids and plays an important role in the development of atherosclerosis. As shown in Figure 5, FABP4 and CD36 interacted closely, further confirming that FABP4 and CD36 were important mediating targets of atherosclerosis. In the atherosclerotic microenvironment, smooth muscle cells, macrophages, and endothelial cells secreted a large number of cytokines that could regulate the expression level of CTSD. The changes in the expression level of CTSD would further lead to the destruction of the dynamic balance of extracellular matrix synthesis and degradation, helping macrophages to become foam cells, mediate the apoptosis of foam cells, and ultimately promote the occurrence of atherosclerosis (Haidar et al. 2006). CTs that interact with CD36 and FABP4 include PPARG, PPARA, CAV1, and NOCA2. PPARG and PPARA, which are key targets for regulating fat synthesis and secretion (Miranda et al. 2024), are also the main targets of existing lipid‐lowering drugs. Studies have shown that Cav‐1 is related to the stability of atherosclerotic plaque (Shu et al. 2023). In summary, hawthorn regulates the formation of foam cells by mediating the targets FABP4 and CD36, as well as its own targets PPARG, PPARA, CTSD, CAV1, and NOCA2.
2. Regulating inflammatory response by CCL3, CCL4, IL1B, and CXCR4

CCL3 and CCL4 are the inflammatory chemokines associated with atherosclerosis. The mRNA of CCL4 and CCL3 was co‐expressed in stimulated cells, and they could stimulate chemotactic T cells, NK cells, macrophages, and other killer cells (Zhang et al. 2018; Simpson et al. 2020). IL1B was a cytokine secreted by monocytes and macrophages that played a role in regulating the body's immune system (Wenjing et al. 2020). The activation of IL‐1B increased the expression of IL‐6 and increased the level of C‐reactive protein. IL‐1B was involved in the occurrence and development of atherosclerosis, and the expression of IL‐1B around coronary atherosclerosis was positively correlated. The chemokine receptor CXCR4 is a specific receptor of chemokine stromal cell‐derived factor‐1 (CXCL12) (Murad et al. 2021). CXCL12 has a strong chemotactic effect on lymphocytes (Korbecki et al. 2021). From Figure 7, the CTs that interacted with CCL3 and CCL4 were IL1A, CXCL10, and IL1B. Therefore, the potential mechanism by which hawthorn regulates inflammatory responses is based on its own targets, IL1B, IL1A, and CXCL10, and their mediated targets, CCL3 and CCL4, which act on immune cells, such as T cells, NK cells, and macrophages, to regulate immune responses.

3 Regulating cell adhesion and migration by targeting CD44 and JUN

CD44 is an adhesion molecule on the cell surface that is primarily involved in cell matrix adhesion. Studies have shown that in patients with coronary heart disease, the mRNA expression of CD44 in the peripheral blood is significantly positively correlated with the Gensini score of the coronary arteries, which could reflect the degree of coronary artery stenosis (Li and Chen 2014). This study indicated that CD44 interacts with collagen and laminin family genes to participate in cell adhesion and migration. In addition, some experiments have confirmed that shear force is involved in atherosclerosis via JNK signalling (JUN‐related pathway), which leads to the secretion of adhesion molecules (VCAM, ICAM, P‐selectin, E‐selectin, etc.) and the recruitment and migration of monocytes and macrophages (Tsukui et al. 2023). As shown in Figure 5, JUN interacted with CTs, such as STAT1, BCL2, ESR2, GSTP1, and RELA. The main CTs that interact with CD44 are SELE, STAT1, and MMP2. It can be seen that the potential mechanism of hawthorn regulating cell adhesion and migration in the atherosclerotic microenvironment was through its own targets, such as STAT1, BCL2, ESR2, GSTP1, RELA, SEL, STAT1, and MMP2, and mediated targets CD44 and JUN, which regulate the formation and fibrosis of plaque and control its stability.

4 Regulating fat synthesis and secretion by targeting PPARG and AREG

PPARG is an important target for regulating fat synthesis and secretion, and the expression and release of AREG genes are induced by various stimuli, including inflammatory lipids. AREG stimulates the proliferation of adipose‐derived stem cells (Han et al. 2023). Based on Figure 9, it can be inferred that hawthorn may regulate fat synthesis and secretion through its own targets, ICAM1 and PPARG, and the mediated target, AREG.

5 Antioxidant stress by targeting MT1X, MT2A, and Hmox1

MT1X and MT2A are regulated by metallothionein, a powerful free radical scavenger with important physiological functions (Heger et al. 2016; Kumar et al. 2024). Numerous studies have shown that excessive reactive oxygen species (ROS) could cause oxidative stress, leading to endothelial dysfunction through a variety of mechanisms that are key factors in the occurrence of atherosclerosis. Metallothionein, an efficient endogenous cytoprotective agent, inhibits the activity of ROS synthase, which directly reduces the production of ROS, reduces oxidative damage to tissues, and participates in the antioxidant stress of atherosclerosis (Cirovic et al. 2024; Sarutipaiboon et al. 2020). HMOX1 is an important gene for oxidative stress that enhances the body's antioxidant capacity, protects cells from oxidative stress damage, and reduces immune responses (Yao et al. 2023). Therefore, hawthorn might realise the role of antioxidant stress in the atherosclerotic microenvironment by targeting HMOX1 as well as the mediated targets MT1X and MT2A.

In conclusion, this study found that the important direct targets of hawthorn in atherosclerosis included IL1B, CTSD, HMOX1, and PPARG. Important indirect (mediated) targets included JUN, CXCR4, CD36, GNLY, FABP4, CCL3, CCL4, MT2A, MT1X, AREG, and CD44. It could be inferred that hawthorn had many effects on atherosclerosis; specifically, it included the following.

1. The control of pathogenic factors, such as the regulation of fat secretion and synthesis, hawthorn regulated the content of body and vascular fat by PPARG and AREG to prevent the formation of hyperlipidemia; thus reducing the possibility of atherosclerosis.
2. Controlling early‐stage atherosclerosis

HMOX1, MT1X, and MT2A could reduce the oxidative damage caused by free radicals to the vascular endothelium; thus, hawthorn could control the early‐stage conditions of atherosclerosis through these three targets.

3 In the disease progression stage, hawthorn reduced the process of phagocytosis of Ox LDL by macrophages and the formation of foam cells by regulating CTSD, CD36, and FABP4, thus preventing foam cells from depositing on the vascular endothelium and forming plaques. Meanwhile, by regulating IL1B, CCL3, CCL4, CXCR4, CCL3, and CCL4, hawthorn could control the immune response of the vascular endothelium and the surrounding microenvironment to prevent the aggravation of the inflammatory response and further deterioration of plaques. In addition, hawthorn could control the progression of plaques by regulating cell adhesion in the vascular endothelium and surrounding microenvironment via JUN and CD44.

## 5 Conclusion

Many plants have multiple targets, and their therapeutic effects on diseases are usually a comprehensive effect of multiple targets. This study proposed a new artificial intelligence method that could be used to systematically reveal the potential mechanisms of food or drugs. The delicious hawthorn could be used not only as food, but also as a drug to prevent the progression of atherosclerosis. This study developed a new research method to discover the potential mechanism of hawthorn on the PVAT microenvironment of atherosclerosis. With artificial intelligence technology, this study integrated the expression levels of hawthorn on multiple targets of atherosclerosis into MTIS, and the landscape and quantitative mapping of the multi‐targets integrated effect of hawthorn at the single cell level has been achieved, and further in‐depth comparative analysis between the non‐atherosclerotic group and atherosclerotic group was performed, such as MTIS comparisons, MTIS‐pseudotime difference analysis, cell communication analysis, immune infiltration analysis, etc. This study will bring new research methods for food analysis, drug analysis, medical mechanism analysis, and life sciences.

## Author Contributions

J.Q.‐y. designed and performed the data experiment, and then analysed data and wrote the manuscript; Z.H.‐y. obtained funding and helped to revise the manuscript. R.T.‐a. and F.X.‐y. performed experimental verification. All authors have read and approved the final manuscript.

## Conflicts of Interest

The authors declare no conflicts of interest.

## Supporting information


**File S1:** pbi70321‐sup‐0001‐FileS1‐S18.zip.

## Data Availability

The data can be obtained by contacting the author with a reasonable request.
